# Superior alleles of *SEED-AGING GENE 9* enhance seed storability and dormancy in rice

**DOI:** 10.1093/nsr/nwag248

**Published:** 2026-04-29

**Authors:** Fulin Zhang, Wenchao Chi, Jiawei Song, Xingjie Zhu, Ping Wang, Yingying Chen, Tengfei Ma, Ziyan Zhu, Qixian Hao, Qingkai Wang, Ye Chen, Kunhao Li, Boliang Lu, Yiwan Wu, Changling Mou, Yunshuai Huang, Hongming Wu, Rong Miao, Qiuyun Lin, Penghui Cao, Shijia Liu, Ling Jiang, Jianmin Wan

**Affiliations:** State Key Laboratory for Crop Genetics and Germplasm Enhancement, Jiangsu Nanjing National Field Scientific Observation and Research Station for Rice Germplasm, Nanjing Agricultural University, Nanjing 210095, China; State Key Laboratory for Crop Genetics and Germplasm Enhancement, Jiangsu Nanjing National Field Scientific Observation and Research Station for Rice Germplasm, Nanjing Agricultural University, Nanjing 210095, China; State Key Laboratory for Crop Genetics and Germplasm Enhancement, Jiangsu Nanjing National Field Scientific Observation and Research Station for Rice Germplasm, Nanjing Agricultural University, Nanjing 210095, China; State Key Laboratory for Crop Genetics and Germplasm Enhancement, Jiangsu Nanjing National Field Scientific Observation and Research Station for Rice Germplasm, Nanjing Agricultural University, Nanjing 210095, China; State Key Laboratory for Crop Genetics and Germplasm Enhancement, Jiangsu Nanjing National Field Scientific Observation and Research Station for Rice Germplasm, Nanjing Agricultural University, Nanjing 210095, China; State Key Laboratory for Crop Genetics and Germplasm Enhancement, Jiangsu Nanjing National Field Scientific Observation and Research Station for Rice Germplasm, Nanjing Agricultural University, Nanjing 210095, China; State Key Laboratory for Crop Genetics and Germplasm Enhancement, Jiangsu Nanjing National Field Scientific Observation and Research Station for Rice Germplasm, Nanjing Agricultural University, Nanjing 210095, China; State Key Laboratory for Crop Genetics and Germplasm Enhancement, Jiangsu Nanjing National Field Scientific Observation and Research Station for Rice Germplasm, Nanjing Agricultural University, Nanjing 210095, China; State Key Laboratory for Crop Genetics and Germplasm Enhancement, Jiangsu Nanjing National Field Scientific Observation and Research Station for Rice Germplasm, Nanjing Agricultural University, Nanjing 210095, China; State Key Laboratory for Crop Genetics and Germplasm Enhancement, Jiangsu Nanjing National Field Scientific Observation and Research Station for Rice Germplasm, Nanjing Agricultural University, Nanjing 210095, China; State Key Laboratory for Crop Genetics and Germplasm Enhancement, Jiangsu Nanjing National Field Scientific Observation and Research Station for Rice Germplasm, Nanjing Agricultural University, Nanjing 210095, China; State Key Laboratory for Crop Genetics and Germplasm Enhancement, Jiangsu Nanjing National Field Scientific Observation and Research Station for Rice Germplasm, Nanjing Agricultural University, Nanjing 210095, China; State Key Laboratory for Crop Genetics and Germplasm Enhancement, Jiangsu Nanjing National Field Scientific Observation and Research Station for Rice Germplasm, Nanjing Agricultural University, Nanjing 210095, China; State Key Laboratory for Crop Genetics and Germplasm Enhancement, Jiangsu Nanjing National Field Scientific Observation and Research Station for Rice Germplasm, Nanjing Agricultural University, Nanjing 210095, China; State Key Laboratory for Crop Genetics and Germplasm Enhancement, Jiangsu Nanjing National Field Scientific Observation and Research Station for Rice Germplasm, Nanjing Agricultural University, Nanjing 210095, China; State Key Laboratory for Crop Genetics and Germplasm Enhancement, Jiangsu Nanjing National Field Scientific Observation and Research Station for Rice Germplasm, Nanjing Agricultural University, Nanjing 210095, China; State Key Laboratory for Crop Genetics and Germplasm Enhancement, Jiangsu Nanjing National Field Scientific Observation and Research Station for Rice Germplasm, Nanjing Agricultural University, Nanjing 210095, China; State Key Laboratory for Crop Genetics and Germplasm Enhancement, Jiangsu Nanjing National Field Scientific Observation and Research Station for Rice Germplasm, Nanjing Agricultural University, Nanjing 210095, China; State Key Laboratory for Crop Genetics and Germplasm Enhancement, Jiangsu Nanjing National Field Scientific Observation and Research Station for Rice Germplasm, Nanjing Agricultural University, Nanjing 210095, China; State Key Laboratory for Crop Genetics and Germplasm Enhancement, Jiangsu Nanjing National Field Scientific Observation and Research Station for Rice Germplasm, Nanjing Agricultural University, Nanjing 210095, China; State Key Laboratory for Crop Genetics and Germplasm Enhancement, Jiangsu Nanjing National Field Scientific Observation and Research Station for Rice Germplasm, Nanjing Agricultural University, Nanjing 210095, China; Zhongshan Biological Breeding Laboratory, Nanjing 210095, China; State Key Laboratory for Crop Genetics and Germplasm Enhancement, Jiangsu Nanjing National Field Scientific Observation and Research Station for Rice Germplasm, Nanjing Agricultural University, Nanjing 210095, China; Zhongshan Biological Breeding Laboratory, Nanjing 210095, China; State Key Laboratory for Crop Genetics and Germplasm Enhancement, Jiangsu Nanjing National Field Scientific Observation and Research Station for Rice Germplasm, Nanjing Agricultural University, Nanjing 210095, China; National Key Facility for Crop Gene Resources and Genetic Improvement, Institute of Crop Science, Chinese Academy of Agricultural Sciences, Beijing 100081, China; Zhongshan Biological Breeding Laboratory, Nanjing 210095, China

**Keywords:** storability, bZIP transcription factors, Late Embryogenesis Abundant proteins, dormancy, domestication, balancing selection

## Abstract

High-quality seeds necessitate both appropriate dormancy to prevent preharvest sprouting and robust storability to preserve germination vigor, which serve as the fundamental guarantee for the successful production and safe storage of crops. As complex and quantitative traits, the improvement of seed quality has been impeded by limited understanding of their regulatory genes. Here, in rice, we identify *SEED-AGING GENE 9* (*SAG9*), encoding a nuclear protein, as a negative regulator underlying the major seed-storability quantitative trait locus *qSS-9*, which also exerts a moderate effect on dormancy. Indeed, SAG9 physically interacts with selected bZIP transcription factors expressed in seeds, inhibiting their transcriptional activation of *Late Embryogenesis Abundant* genes—revealing a shared genetic pathway coordinating dormancy and storability. This has driven the differential local adaptation of *SAG9* distinct haplotypes, thereby promoting balancing selection during rice domestication. Notably, the elite *SAG9* haplotype *Hap−* has been largely lost during domestication, which is one of the causes for the reduction in seed dormancy and storability of modern varieties. Furthermore, strong-dormancy alleles (*SD1, SDR3.1, Sdr4, SD6*, and *Rc*) enhance storability and show additive effects with *SAG9 Hap−*. Diverse pyramiding combinations of these genes across regions reflect synergistic adaptation to environmental or breeding demands. Collectively, our study elucidates the molecular and domestication basis of seed quality traits, providing targets for precision breeding in modern rice varieties.

## INTRODUCTION

Seed vigor, which peaks following physiological after-ripening and subsequently declines irreversibly during storage, is critical for crop establishment [1,2]. This trait governs germination, seedling emergence and stress tolerance, and ultimately grain yield. The manifestation of seed vigor is constrained by dormancy. This constraint also facilitates resistance to preharvest sprouting (PHS), which occurs frequently in rice-growing regions with high temperatures and precipitation and severely affects grain yield and quality [3,4]. Therefore, appropriate seed dormancy is crucial for agricultural production. On the other hand, seed vigor is rapidly lost under high-temperature and high-humidity conditions, which requires strong storability to prevent [5–7]. Statistics show that improper rice storage in China could result in an annual yield loss of over 3% [8], and PHS causes an annual economic loss of one billion worldwide [3], making these serious problems in terms of both global agricultural production and the economy, especially against the backdrop of global warming [9–12], motivating the identification of key genetic regulators governing these traits.

Accumulating evidence indicates potential overlapping regulatory pathways governing seed storability and dormancy, particularly within the abscisic acid (ABA) signaling pathway. The transcription factor ABI5 enhances storability in legumes and maize (*Zea mays*), while its rice ortholog promotes dormancy [13–15]. The bZIP23-mediated expression of *PER1A* and *Rab16A* positively regulates seed storability and dormancy, respectively [5,16]. The ABA-signaling markers *EM1* and *LEA3*, homologs of genes that positively regulate storability in *Arabidopsis thaliana* [17], act as positive regulators of dormancy in rice [15]. With mutants deficient in ABA signaling or perception, such as *abi3* (ABA-insensitive), *aba1* (ABA-deficient), and *dog1* (*DELAY OF GERMINATION 1* mutant) having a complete loss of dormancy and compromised longevity (storability) [18–23]. Furthermore, antioxidant systems also likely link storability and dormancy by modulating seed aging and dormancy release [5,22,24,25]. Despite these potential connections and shared regulatory components, a direct molecular pathway that explicitly coordinates seed storability and dormancy has not been fully elucidated.

Trade-offs between evolutionary gain and loss are prevalent in nature, which often lead to the balanced selection of genes [26,27]. A textbook example of an evolutionary trade-off and balancing selection is human sickle-cell disease, the most common monogenic genetic disorder, but can also provide remarkable protection against severe malaria [28–30]. In plants, the evolution of plant resistance to pathogens often comes at the costs of reduced growth, driving haplotypes with growth superiority or disease-resistance superiority selected in distinct regions [31,32]. Domestication-driven selection for strong-vigor seeds has led to a substantial loss of dormancy [15,33,34], which may also result in the loss of storability. Whether there is a trade-off between seed storability and vigor, and how this trade-off restricts the domestication of storability, remain unaddressed.

Here, we identify *SEED-AGING GENE 9* (*SAG9*) as a regulator coordinating seed storability and dormancy in rice. SAG9 represses activation capacity of several bZIP transcription factors expressed in seeds, thereby suppressing their activation of *Late Embryogenesis Abundant* (*LEA*) genes—uncovering a shared regulatory module governing both traits. An elite *SAG9* haplotype (*Hap−*) with nuclear-localization deficiency enhances storability while maintaining moderate dormancy, making it a valuable breeding target. Loss of *SAG9* enhances longevity by ≥8 months but delays initial germination, reflecting a trade-off between seed storability and vigor. Indeed, the domestication for strong vigor has restricted the improvement of seed storability and dormancy, particularly in modern varieties from southern China. Furthermore, our work has summarized the domestication trajectories of genes related to dormancy and storability (*SAG9, Sdr4*, etc.), providing a theoretical basis and candidate targets for seed quality improvement.

## RESULTS

### A natural allele of *qSS-9* in Nipponbare acts as a dominant-negative regulator of seed storability

Fine mapping previously confined the major seed-storability quantitative trait locus (QTL) *qSS-9* to a 147-kb interval on chromosome 9 containing seven annotated genes (including *Os09g0368101, Os09g0368200, Os09g0368500, Os09g0368900, Os09g0369000*, and *Os09g0369400*) and five hypothetical proteins (including *Os09g0369500*) [35]. Independent mapping also delimited this locus (*qLG-9*) to a 30-kb region [36]. Comparative analysis identified *Os09g0369400* and *Os09g0369500* as overlapping candidates. *Os09g0369500* expresses two transcript isoforms and is strongly and specifically expressed in seeds during seed maturation and artificial-aging stage in both the weak-storability cultivar Nipponbare and strong-storability chromosome single-segment substitution line SL36 (derived from Nipponbare × Kasalath) (Fig. S1a–e). Sequencing did not detect polymorphisms in *Os09g0369400* between Nipponbare and SL36, but four variants were identified in *Os09g0369500*: two intronic single-nucleotide polymorphisms (SNPs, at position +2028 C/T and at +2154 A/G) and two exonic insertions/deletions (InDels, +3029 CTCG/G causing a deletion of leucine at +222; and at +3964 GA/− resulting in premature translation termination) (Fig. S1d and f). *Os09g0369500* was therefore prioritized for verification as the causal gene for *qSS-9/qLG-9*.

**Figure 1.**
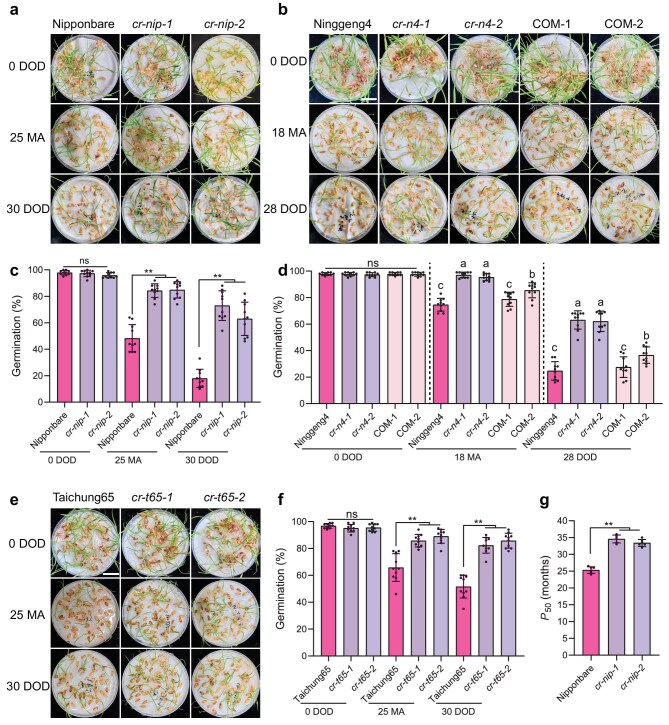
*Os09g0369500* negatively regulates seed storability in rice. Germination performance for cv. Nipponbare (a), Ninggeng4 (b), and Taichung65 (e), respective pair mutant of *Os09g0369500* alleles per accession and transgenic complementation lines (COM-1, -2) in the Ninggeng4 mutant background. Among them, *Os09g0369500* sequences in Ninggeng4 and Taichung65 are identical to the Nipponbare genome. DOD, days of deterioration; MA, months of aging. Photographs were taken after 7–10 days of imbibition. Scale bars: 2 cm. Germination of seeds in Nipponbare (c), Ninggeng4 (d), and Taichung65 (f) backgrounds, respective pairs of mutant alleles and transgenic complementation lines (COM-1, -2) in the Ninggeng4 mutant background. *n* = 10 replicates and each biological replicate comprised three independent technical replicates. (g) *P*_50_ (the time for germination percentage to decrease to 50%) for Nipponbare and two mutant alleles. *n* = 6 replicates, each biological replicate comprised three independent technical replicates. Data are presented as mean ± standard deviation (SD). Statistical analysis were performed using one-way analysis of variance (ANOVA) followed by Duncan’s new multiple range tests for mutants or complementation lines versus respective WTs within each time point in panels (c) and (f), using one-way ANOVA followed by Duncan’s new multiple range tests within each time point in panel (d), and using one-way ANOVA followed by Duncan’s new multiple range tests for mutants versus Nipponbare in panel (g). ns, nonsignificant differences. Singular asterisk or different letters indicate a statistically significant difference at *P < 0.05; two asterisks, **P < 0.01.

To validate the function of *Os09g0369500*, CRISPR–Cas9 gene editing was used to generate two mutant alleles (assigned −1 and −2) in each of the five backgrounds: Nipponbare (*cr-nip-1/-2*), Ninggeng4 (*cr-n4-1/-2*), SL36 (*cr-sl-1/-2*), Kasalath (*cr-kas-1/-2*), and N22 (*cr-n22-1/-2*) (Fig. S1g and h). All mutants and wild-type (WT) controls showed comparable initial germination (>90%), indicating no effect on germination competence (Fig. 1a–d and Fig. S2a–f). After ambient storage or accelerated aging, *cr-nip-1/-2* and *cr-n4-1/-2* mutants had statistically significantly higher germination rates than respective WTs (Fig. 1a–d), whereas *cr-sl-1/-2, cr-kas-1/-2*, and *cr-n22-1/-2* showed no storability difference from controls (Fig. S2a–f). Complementation of *cr-n4-1* with *Os09g0369500-01* driven by its native promoter restored the storability defect to the WT situation (Fig. 1b and d). Furthermore, mutations in flanking genes (*Os09g0368900, Os09g0369000* and *Os09g0369400*) also obtained by gene editing conferred no obvious phenotypic effect (Fig. S2g–m). These results confirm *Os09g0369500* is the causal gene at *qSS-9*, and was designated as *SAG9*, which acts as a negative regulator in Nipponbare and Ninggeng4 but is seemingly functionally impaired in SL36, Kasalath, and N22 backgrounds. Notably, knockout of *SAG9* in the Nipponbare background extended seed longevity by ≥8 months (Fig. 1g). Heterozygotes from Nipponbare × *cr-nip-1* crosses had storability identical to Nipponbare (Fig. S1i), demonstrating dominant negative regulation by the Nipponbare allele.

Given two *SAG9* transcript isoforms are expressed, we targeted the longer transcript (*Os09g0369500-01*) in the Taichung65 (*cr-t65-1/-2*) background using a specific single guide RNA (sgRNA) site in its third exon (Fig. S1j and k). Enhanced storability in *cr-t65-1/-2* versus WT confirmed *Os09g0369500-01* as the functional transcript (Fig. 1e and f).

### A deletion impairs SAG9^Kas^ nuclear localization and function

**Figure 2.**
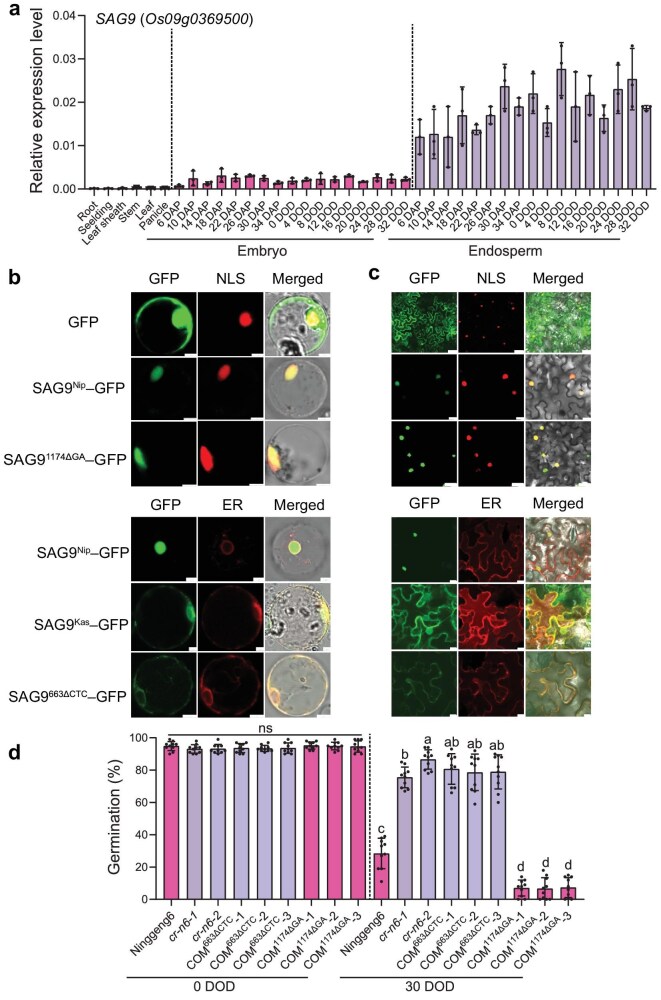
A deletion variant impairs SAG9 nuclear localization and function in cv. Kasalath. (a) *SAG9* expression pattern in Nipponbare. DAP, days after pollination; DOD, days of deterioration. Data are presented as mean ± SD of *n* = 3 replicates. Each replicate comprised three independent technical replicates. Subcellular localization of SAG9–GFP isoforms expressed in rice protoplasts (b) and tobacco leaves (c). NLS, nucleus localization signal using D53–mCherry [72]; ER, endoplasmic reticulum localization signal using mCherry–KDEL; SAG9^Nip^, cloned from Nipponbare; SAG9^Kas^, cloned from Kasalath; SAG9^663ΔCTC^, SAG9^Nip^ with a deletion of ‘CTC’ at position +663; SAG9^1174ΔGA^, SAG9^Nip^ with a deletion of ‘GA’ at position +1174. Scale bars: 5 μm in panel (b), 10 μm in panel (c). (d) Germination for WT Ninggeng6, two *sag9* mutants *cr-n6-1/-2*, and transgenic complementation lines. COM^663ΔCTC^, *cr-n6-2* complemented with native-promoter-driven *SAG9^c.663^^-^^665delCTC^*; COM^1174ΔGA^, *cr-n6-2* complemented with native-promoter-driven *SAG9^c.1174_1175delGA^. n* = 10 replicates, each biological replicate comprised three independent technical replicates. Data are presented as mean ± SD. Statistical analysis was performed using one-way ANOVA followed by Duncan’s new multiple range tests within each time point. ns, nonsignificant difference. Different letters above the bars indicate a significant difference at *P < 0.05.

*SAG9* is predicted to encode a hypothetical protein comprised of 397 amino acids, without known domains. Spatial expression analysis using an *SAG9_pro_: RUBY* reporter cassette in stable*-*transgenic cultivar (cv.) Nipponbare plants (Fig. S3a) and qRT-PCR (Fig. 2a) revealed predominant *SAG9* messenger RNA (mRNA) accumulation in endosperm, moderate levels in embryos, indicating a seed-specific role. Phylogenetic analysis indicates *SAG9* orthologs are exclusive to Poaceae, suggesting late evolutionary emergence (Fig. S3b).

Confocal microscopy demonstrated nuclear localization of SAG9^Nip^–GFP but cytoplasmic distribution of SAG9^Kas^–GFP, together with partial endoplasmic reticulum colocalization for the latter (Fig. 2b and c). To identify causal variants affecting subcellular localization, we generated two site-directed mutants: SAG9^663ΔCTC^–GFP (deletion of CTC at nucleotide positions 663–665 of CDS) and SAG9^1174ΔGA^–GFP (deletion of GA at positions 1174–1175 of CDS). SAG9^663ΔCTC^–GFP recapitulated the cytoplasmic localization of SAG9^Kas^, while SAG9^1174ΔGA^–GFP retained exclusive nuclear localization (Fig. 2b and c), indicating that deletion of CTC is necessary and sufficient for nuclear exclusion.

To assess functional consequences, we complemented the Ninggeng6 *sag9* mutant (*cr-n6-2*, with enhanced storability vs WT Ninggeng6; Fig. 2b and c, and Figs S1f and g and S3c) with two constructs driven by the native promoter: *SAG9^c.663–665^^Δ^^CTC^* (COM^663ΔCTC^) or *SAG9^c.1174–1175^^Δ^^GA^* (COM^1174ΔGA^). After accelerated aging, *COM^1174ΔGA^* transgenic lines 1–3 showed a statistically significant reduction in storability versus *cr-n6-2*, whereas *COM^663ΔCTC^* lines 1–3 bore no difference to *cr-n6-2* (Fig. 2b and c and Fig. S3c). This confirms that deletion of CTC abolishes SAG9’s negative regulatory function by disrupting nuclear localization.

### Rice SAG9 inhibits activity of selected bZIP transcription factors to attenuate seed storability

Toward resolving the molecular mechanism by which nuclear SAG9^Nip^ acts in seed-longevity regulation, we first confirmed its apparent lack of transcriptional-activation activity in yeast and rice protoplasts (Fig. S4).

**Figure 3.**
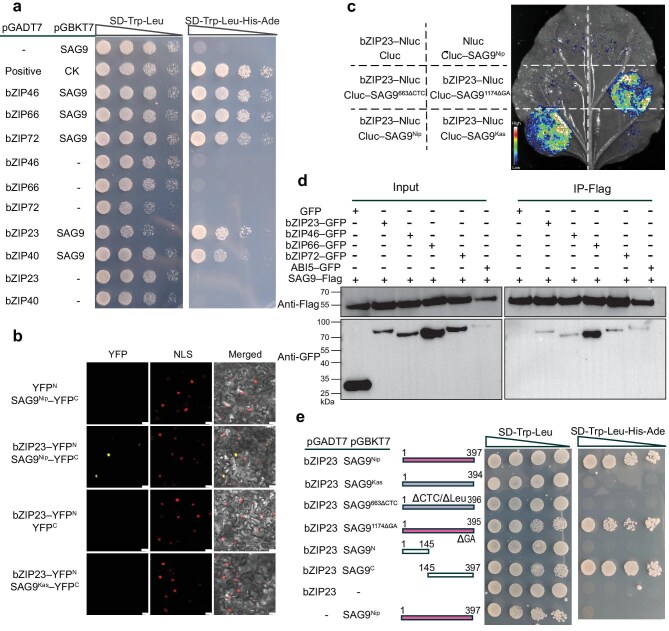
Rice SAG9 physically interacts with several bZIP transcription factors. (a) Y2H assay of physical interactions between SAG9 and selected bZIP transcription factors. Assay of physical interactions between SAG9 and bZIP23 by BiFC (b) and split-luciferase complementation (c) in tobacco leaves. Scale bars: 10 μm. (d) Co-IP of the interaction between SAG9–FLAG and bZIP–GFP in rice protoplasts. (e) Y2H of the interaction between SAG9 isoforms and bZIP23. SAG9^Nip^, cloned from Nipponbare; SAG9^Kas^, cloned from Kasalath; SAG9^663ΔCTC^, SAG9^Nip^ with a deletion of ‘CTC’ at position +663; SAG9^1174ΔGA^, SAG9^Nip^ with a deletion of ‘GA’ at position +1174; SAG9^N^, amino acids 1–145 of SAG9^Nip^; SAG9^C^, amino acids 145–397 of SAG9^Nip^.

To screen for potential interacting proteins, using SAG9^Nip^ as the bait, we performed a yeast two-hybrid (Y2H) screening assay using a complementary DNA (cDNA) library from N22 seeds at the maturation stage, and identified three transcription factors: bZIP46 (*Os06g0211200*), bZIP66 (*Os08g0472000*), and bZIP72 (*Os09g0456200*). These bZIP transcription factors cluster within the evolutionary conserved bZIP class-E subfamily [37], which includes known storability regulators *bZIP23, bZIP42*, and *bZIP10*/*ABI5* [5,13,14]. Thus, we hypothesize that bZIPs in this subfamily may interact with SAG9. Subsequently, we selected class-E bZIPs, which are highly expressed in seeds, and confirmed that ABI5/bZIP10, bZIP23, bZIP40, bZIP46, bZIP66, and bZIP72 interact with SAG9 using Y2H (Fig. 3a), bimolecular fluorescence complementation (BiFC; Fig. 3b and Fig. S5a and d), luciferase complementation imaging (Fig. 3c and Fig. S5e), and co-immunoprecipitation (Co-IP; Fig. 3d). In addition, domain mapping using SAG9 truncations (SAG9^N^ and SAG9^C^, amino acids 1–145 and 145–397 of SAG9^Nip^, respectively) and site-directed variants (SAG9^663ΔCTC^ and SAG9^1174ΔGA^, deletion of CTC and GA at nucleotide 663 and 1174 of CDS, respectively) demonstrated its CTC-dependent interaction via the C-terminal domain (Fig. 3c–e and Fig. S5e–h).

To verify the role of bZIPs in seed storability, we performed accelerated-aging assays and found that *bZIP10*/*ABI5* mutants (*osabi5-1/-2* in Nipponbare background; *abi5-1/-2* in ZH11 background), *bZIP66* and *bZIP72* double mutants (*crbzip72/crtrab1-1* in Nipponbare background), and *bZIP46* knockout lines (*bzip46-1/-2* in Ninggeng7 background) showed impaired seed storability versus their respective WT, whereas *bZIP10/ABI5* overexpression (OE-1/-2 in Nipponbare background; OE-ABI5-1/2 in ZH11 background) showed enhanced storability (Fig. S6). These results confirm that *bZIP46, bZIP66, bZIP72*, and *bZIP10/ABI5* are positive regulators of rice seed storability as previously reported *bZIP23* and *bZIP42* [5].

To verify the genetic relationship between *SAG9* and *bZIP10/ABI5*, we obtained two bZIP10/ABI5 mutant alleles in the N22 background (*n22-abi5-1/-2*) that contains a nonfunctional SAG9 allele (Fig. S7a and b). The seed storability of *n22-abi5-1/-2* decreased significantly than N22 controls (Fig. S7e and f), with the magnitude of 31.17%–32.83%, greater than that in Nipponbare background (12.7%–13.1%; Fig. S6a and b) and ZH11 background (10.3%–16.4%; Fig. S6g and h), suggesting that SAG9^Nip^ may partially interfere with the effect of bZIP10/ABI5.

**Figure 4.**
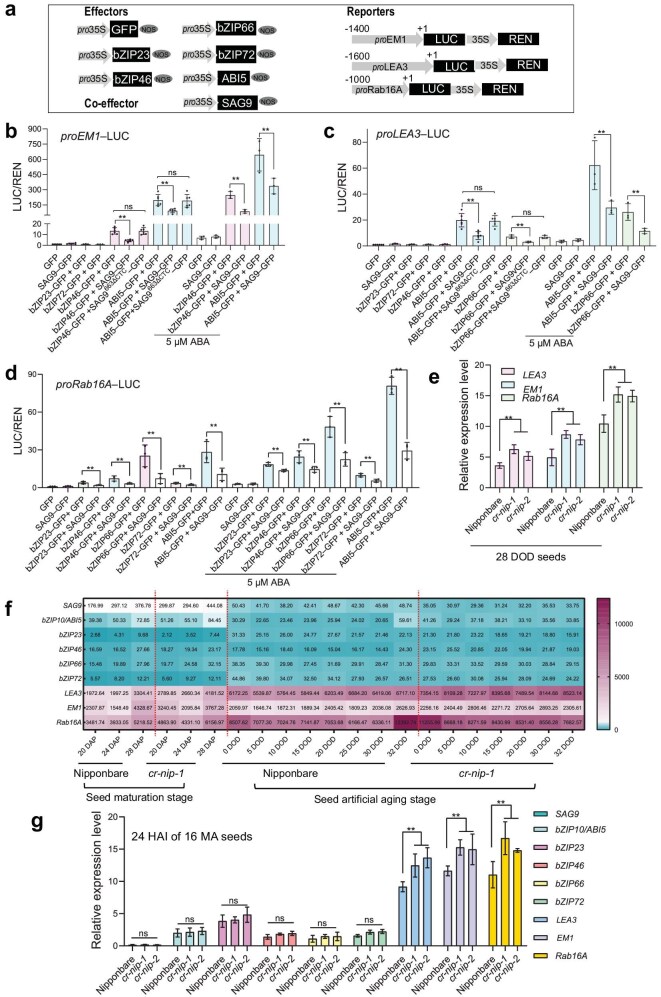
SAG9 inhibits the activation activity of selected bZIP transcription factors. (a) Effector (left) and reporter (right) constructs used in dual-luciferase reporter assays carried out in rice protoplasts. Numbers above reporter constructs depict lengths in base pairs of the respective promoter regions assayed. LUC/REN values for assays reporting activation of *EM1* (b), *LEA3* (c), and *Rab16A* (d) promoters in the absence or presence of 5 μM ABA using constructs shown in panel (a). *n* = 3 replicates for ABA treatments or *n* = 6 for untreated controls, with each biological replicate comprised three independent technical replicates. SAG9^663ΔCTC^, SAG9 with a deletion of ‘CTC’ at position +663. (e) *LEA3, EM1*, and *Rab16A* expression in WT cv. Nipponbare and mutant seeds at 28 DOD determined by qRT-PCR. *n* = 3, each biological replicate comprises independent technical replicates. (f) Transcript levels (TPM) of *SAG9, bZIPs*, and *LEAs* in WT and *cr-nip-1* seeds at maturation and artificial-aging stages. DAP, days after pollination; DOD, days of deterioration; TPM, transcripts per million. (g) qRT-PCR analysis of *SAG9, bZIPs*, and *LEAs* at 24 HAI. The seeds (harvested at Nanjing, 2024) utilized in this experiment had undergone 16 months of natural storage prior to imbibition. Data are presented as means ± SD. Statistical analysis were performed using one-way ANOVA followed by Duncan’s new multiple range tests versus respective effector without cotransform of SAG9–GFP in panels (b)–(d) and using one-way ANOVA followed by Duncan’s new multiple range tests versus Nipponbare in panels (e) and (g). HAI, hours after seed imbibition; ns, nonsignificant difference. Singular asterisk indicate a significant difference at *P < 0.05; two asterisks, **P < 0.01.

Given bZIP10/ABI5, bZIP23, bZIP46, bZIP66, and bZIP72 are typical transcription factors, their physical interactions with SAG9 may inhibit their transcriptional-activation activity, with known roles for some of these bZIPs in regulating expression of genes acting in rice seed dormancy and storability [5,15,16]. To determine whether these known genetic pathways are associated with SAG9-regulated seed storability, we performed dual-luciferase reporter assays and saw that bZIP23 and bZIP10/ABI5 could activate the promoter of *PER1A* (Fig. S8a and b); bZIP46 and bZIP10/ABI5 could activate the *EM1* promoter (Fig. 4a and b); bZIP66 and bZIP10/ABI5 could activate the *LEA3* promoter (Fig. 4a and c); and bZIP23, bZIP46, bZIP66, bZIP72, and bZIP10/ABI5 could activate the *Rab16A* promoter (Fig. 4a and d). Co-expression of SAG9, but not SAG9^663ΔCTC^, downregulated the activation of the *LEA3, EM1*, and *Rab16A* promoters (Fig. 4a–d) but spared the activation of *PER1A* (Fig. S8a and b), suggesting SAG9 could partially suppress the activation of *LEA3, EM1*, and *Rab16A* promoters by bZIPs. Under ABA treatment, SAG9 still retained the ability to suppress the activation of *LEAs* by bZIPs, indicating that SAG9 functions as a repressor of the ABA signaling pathway. Consistent with this, *EM1, LEA3*, and *Rab16A* were upregulated in *sag9* seeds compared to Nipponbare (Fig. 4e), while *PER1A* remained unchanged (Fig. S8c), suggesting that SAG9 can suppress the expression of *EM1, LEA3*, and *Rab16A* in seeds.

*EM1, LEA3*, and *Rab16A* have been reported to be regulated by *bZIP10/23/46/66/72* and to positively regulate seed dormancy in rice [15,16,38–41]. To assess their impact on seed storability, we performed artificial-aging assays and saw that storability was reduced in *em1-1, em1-2, lea3-1*, and *lea3-2* mutants compared to the WT ZH11, whereas overexpression lines had enhanced storability (Fig. S6g and h). These findings indicated that *EM1* and *LEA3* function as positive regulators of seed storability, with SAG9 negatively regulating seed storability by directly suppressing activity of bZIPs and in turn *LEA* expression. Supporting this model, artificial aging induced accumulation of malondialdehyde (a biomarker of lipid peroxidation) in Nipponbare seeds compared to *sag9* seeds (Fig. S8d). This indicates more severe oxidative damage in the WT background. Furthermore, peroxidase activity remained unchanged (Fig. S8e and f), suggesting that the SAG9-mediated reduction in antioxidant capacity primarily stems from impaired nonenzymatic protection mechanisms associated with LEAs. These mechanisms are known to include cellular nonenzymatic antioxidant activity and the formation of glassy cytoplasm, which can restrict molecular mobility, reduce cellular metabolic activity, and stabilize cellular components [6,17].

To investigate the temporal dynamics of the SAG9–bZIPs–LEAs pathway, we performed RNA sequencing analysis on seeds during maturation and artificial aging, and conducted quantitative real-time polymerase chain reaction (qRT-PCR) assays on aged seeds during imbibition. The results showed that *SAG9, bZIPs*, and *LEAs* were constitutively expressed across these stages (Fig. 4f and g). Notably, *LEAs* exhibited higher expression levels in *sag9* knockout lines compared to Nipponbare (Fig. 4f and g). Collectively, these findings indicate that the SAG9–bZIPs–LEAs pathway functions throughout seed maturation, storage, and imbibition stages.

### *SAG9* negatively regulates seed dormancy in rice

**Figure 5.**
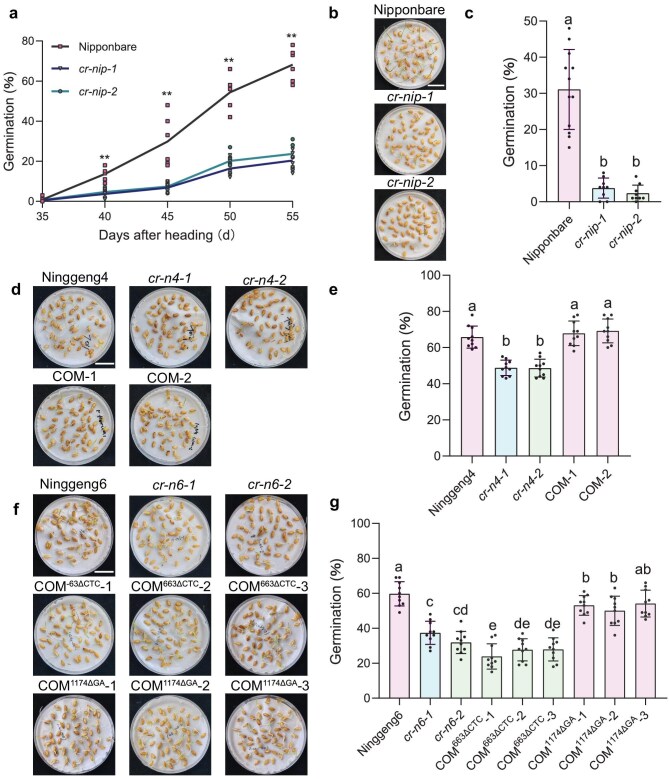
*SAG9* positively regulates seed dormancy in rice. (a) Germination of Nipponbare and mutant seeds harvested at 35–55 DAH. This picture presents the germination statistics of seeds harvested in Nanjing in 2021. Data are presented as mean ± SD of *n* = 6, each biological replicate comprised three independent technical replicates. Germination performance of fresh-harvested seeds from cv. Nipponbare (b), Ninggeng4 (d), and Ninggeng6 (f) backgrounds and respective mutant alleles. Scale bars: 2 cm. Germination of freshly harvested seeds from Nipponbare (c), Ninggeng4 (e), and Ninggeng6 (g) backgrounds, respective mutant alleles and transgenic complementation lines in the Ninggeng4 background. COM, *cr-n4-1* complemented with native-promoter-driven *Os09g0369500-01*; COM^663ΔCTC^, *cr-n6-2* complemented with native-promoter-driven *SAG9^c.663–665ΔCTC^*; COM^1174ΔGA^, *cr-n6-2* complemented with native-promoter-driven *SAG9^c.1174_1175ΔGA^*. Data are presented as mean ± SD of *n* = 10, each biological replicate comprised three independent technical replicates. Statistical analysis was performed using one-way ANOVA followed by Duncan’s new multiple range tests. Singular asterisk and different letters indicate a statistically significant difference at *P < 0.05; two asterisks, **P < 0.01.

Given the established roles of *bZIP10/ABI5, bZIP23*, bZIP46, *bZIP66, bZIP72*, and *LEAs* in ABA signaling and regulation of dormancy in rice [16,42–52], we investigated the potential role of *SAG9* in regulating seed dormancy. Seeds harvested at 35 days after heading (DAH) were dormant, as indicated by minimal (i.e. 0.5%) germination (Fig. 5a). As harvest time was delayed, the germination percentages of Nipponbare increased more rapidly than that of the *sag9* mutants (*cr-nip-1* and *cr-nip-2)*, reaching nearly 30% at 45 DAH. In contrast, *sag9* mutants maintained a lower germination rate of <10% at this stage (Fig. 5b). This enhanced-dormancy phenotype in *sag9* mutants was consistently observed in the Ninggeng4 and Ninggeng6 backgrounds, with mutants having lower germination rates compared to their respective WTs, and dormancy was restored in transgenic complementation lines (Fig. 5d–g). Similar to seed-storability results, no statistically significant differences in dormancy were detected between *sag9* knockout mutants and their corresponding WT in the SL36, N22, or Kasalath backgrounds (Fig. S9a–f). Collectively, these results demonstrate that *SAG9* acts as a negative regulator of seed dormancy.

ABA and Gibberellin (GA) are two key hormones that antagonistically regulate seed dormancy and germination [3,53]. Hormone-response assays revealed that Nipponbare is more sensitive to GA_3_ but has reduced sensitivity to ABA compared to *sag9* mutants (Fig. S9g and h). Comparable endogenous ABA levels between Nipponbare and mutants indicated that *SAG9* modulates ABA signal transduction rather than its biosynthesis (Fig. S9j). Collectively, these observations establish that the SAG9–bZIPs–LEAs pathway physiologically regulates both seed dormancy and storability, and this mechanistic connection aligns with the positive dormancy–storability correlation observed in natural rice populations (Fig. S9k), suggesting potential for concurrent genetic improvement of both traits.

To investigate the effect of *SAG9* on seed vigor during storage, we thoroughly examined seed vigor of Nipponbare and *sag9* mutants and found that *SAG9* confers higher vigor during brief storage (1–14 months) but lower vigor during prolonged storage (15–36 months) (Fig. S9i), revealing a trade-off between seed vigor and storability. This result infers that domestication-driven selection for strong vigor may constrain the improvement of storability, analogous to its constraining effect on dormancy [15,33,34].

### *SAG9* natural variation is associated with differences in seed storability and dormancy

To identify causal genetic variations in *SAG9* underlying seed storability and dormancy variation, we analysed its sequence in the 3000 Rice Genomes Project [54–56], revealing seven coding-region InDels that define 20 haplotypes (*Hap1–20*, Fig. S10a). Based on the assumption that *SAG9* is functional in Nipponbare and Ninggeng6 but nonfunctional in Kasalath (Figs 1c and 2d and Fig. S2c and d), InDel3 (CTCG/G; differing between Nipponbare and Kasalath by CTC deletion at 663 of Kasalath CDS and leucine deletion at 222 of Kasalath amino acid) was therefore prioritized as the major functional variant, contrasting with InDel 1, 2, and 5–7 that differ between Nipponbare and Ninggeng6 (Fig. S10b). Consistent with this, a strong association signal was observed between InDel3 and seed storability (Fig. S10c). Consequently, haplotypes were classified into three functional groups based on InDel3 status: *Hap+* (functional, e.g. *Hap1–10*), *Hap−* (loss-of-function, e.g. *Hap11–17*), and *HapX* (uncertain function, because it is unclear whether the deletion at InDel4 or InDel5 will affect the function of SAG9, e.g. *Hap18–20*) (Fig. S10a).

Evolutionary analysis showed that wild rice exclusively harbors only *Hap+* (*Hap1* and *Hap10*), indicating these are ancestral haplotypes (Fig. S10a and g). Haplotypes were differentially distributed across subspecies with 91.36% *Hap−* in *Aus* (Fig. S10d) and 75.82% *Hap−* in *Japonica* (Fig. S10e), wherein *Hap+* was mainly concentrated in accessions from high-latitude zones (Fig. S10g), reflecting its potential high-latitude adaptive role. Moreover, 73.64% of *Indica* accessions are *Hap+* (Fig. S10f), with *Hap−* mainly enriched in accessions from tropical areas, reflecting its potential high-temperature and high-humidity adaptive role (Fig. S10h). Haplotype-network reconstruction revealed that *Hap−* likely originated from *Hap+* (i.e. *Hap1*) via multiple independent deletion events (Fig. S10i).

**Figure 6.**
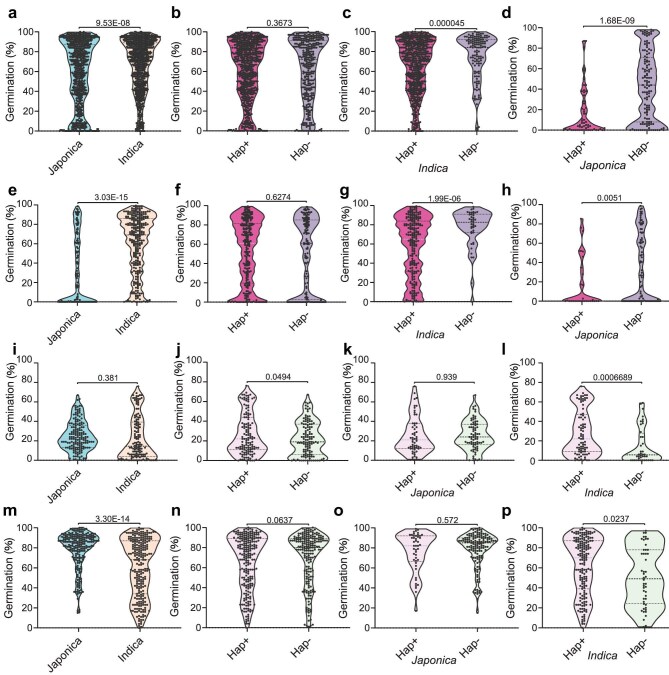
Natural variation in rice *SAG9* is associated with differences in seed storability and dormancy. Comparison of seed storability between *Japonica* and *Indica* subgroups (a), between *SAG9* haplotypes *Hap+* and *Hap−* (b), between *SAG9* haplotypes *Hap+* and *Hap−* in the *Indica* subgroup (c), and between *SAG9* haplotypes *Hap+* and *Hap−* in the *Japonica* subgroup (d) based on 2 years of artificial-aging assays for 691 varieties listed in Table S2. Comparison of seed storability between *Japonica* and *Indica* subgroups (e), between *SAG9* haplotypes *Hap+* and *Hap−* (f), between *SAG9* haplotypes *Hap+* and *Hap−* in the *Indica* subgroup (g), and between *SAG9* haplotypes *Hap+* and *Hap−* in the *Japonica* subgroup (h) based on published artificial-aging data provided in Table S6. Comparison of seed dormancy between *Japonica* and *Indica* subgroups (i), between *SAG9* haplotypes *Hap+* and *Hap−* (j), between *SAG9* haplotypes *Hap+* and *Hap−* in the *Indica* subgroup (k), and between *SAG9* haplotypes *Hap+* and *Hap−* in the *Japonica* subgroup (l) based on published seed dormancy data provided in Table S7. Comparison of seed dormancy between *Japonica* and *Indica* subgroups (m), between *SAG9* haplotypes *Hap+* and *Hap−* (n), between *SAG9* haplotypes *Hap+* and *Hap−* in the *Indica* subgroup (o), and between *SAG9* haplotypes *Hap+* and *Hap−* in the *Japonica* subgroup (p) based on additional published seed-dormancy data provided in Table S3. Dotted lines denote the 25th percentile, the median, and the 75th percentile. Statistical analysis was performed using two-tailed Student’s *t* tests and numbers annotated for each comparison are the exact *P* values.

To establish genotype*–*storability associations, we conducted artificial-aging assays on a diverse panel of 691 rice accessions. Initial assessment revealed higher seed storability in *Indica* (predominantly *Hap+*) than *Japonica* accessions (predominantly *Hap−*) (Fig. 6a), contradicting the expected superior storability of *Hap−*. This discrepancy indicated that subspecies divergence, rather than *SAG9* variation, drives the storability difference between *Indica* and *Japonica*. To avoid the interference of subspecies differences (Fig. 6b), we performed stratified analyses within each subspecies. Consistent with *SAG9*’s functional role, *Hap−* accessions had higher storability than *Hap+* in both *Indica* (Fig. 6c) and *Japonica* (Fig. 6d). This allele effect was further validated using an independent storability dataset [57], showing concordant directional effects in both subgroups (Fig. 6e and f). Collectively, these results demonstrate that natural variation in *SAG9* modulate seed storability, with the predominant retention of *Hap−* in *Japonica* reflecting its selective advantage for stronger seed storability.

To establish an association between genotype and dormancy, we analysed two previous, independent seed-dormancy datasets [33,58]. Despite seed-dormancy comparison between *Indica* and *Japonica* subgroups (Fig. 6i and m) and between *Hap+* and *Hap−* accessions (Fig. 6j and n) showing discordant trends across these two datasets, both datasets robustly demonstrated that *Hap−* accessions had higher dormancy than *Hap+* in the *Indica* subgroup (Fig. 6l and p). In contrast, no statistically significant dormancy difference between haplotypes was observed in *Japonica* (Fig. 6k and o). These results indicate that natural variation in *SAG9* modulates seed dormancy specifically in *Indica*, with the predominant retention of *Hap+* reflecting its selective advantage for faster germination.

Collectively, natural variation in *SAG9* contributes to both seed storability and dormancy. *SAG9* functions as an equalizer of these two traits in natural populations, with *Hap+* predominantly retained in *Indica* to reduce dormancy for accelerated germination and *Hap−* favored in lower-storability *Japonica* to enhance storability.

Notably, *Hap−*, a superior haplotype conferring stronger dormancy and storability, has been gradually neglected in modern breeding processes, particularly in southern China, where only 2 of 654 sequenced modern *Japonica* accessions and 0/627 modern *Indica* accessions harbor *SAG9 Hap−* (Fig. S11 and Table S8). Knockout of *SAG9* in modern varieties (Ninggeng7, Asominori, Wuyugeng3: all in *Hap1*; Ninggeng8: in *Hap3*) enhanced seed storability (Fig. S12) and dormancy (Fig. S13) without compromising major agronomic traits (Fig. S14), demonstrating its potential for breeding applications. Furthermore, compared with related WT, the germination rates of the knockout lines in the Nipponbare, Ninggeng4, Ninggeng6, Ninggeng7, Ninggeng8, Taichung65, and Wuyugeng3 backgrounds were reduced by 33.0%–34.5% (Fig. 5c), 17.0%–17.2% (Fig. 5e), 22.3%–27.8% (Fig. 5g), 13.0%–13.9% (Fig. S13f), 8.4%–9.1% (Fig. S13h), 16.0%–19.0% (Fig. S13i), and 28.2% (Fig. S13j) during the dormancy stage, respectively, while the germination rates of these lines were increased by 45.0%–55.0% (Fig. 1c), 37.4%–38.4% (Fig. 1d), 47.1%–58.2% (Fig. 2d), 27.0%–33.2% (Fig. S12e), 17.9%–19.9% (Fig. S12b), 30.7%–33.9% (Fig. 1f), and 53.4% (Fig. S12h) after artificial aging, respectively. These findings demonstrate that the effect of *SAG9* on seed dormancy is moderate in comparison with its prominent effect on seed storability, and *SAG9* could be the valuable target for breeding rice varieties with enhanced storability and moderate dormancy. Given the high ‘GC’ content in the genomic regions flanking ‘CTC’, we provide a pair of specific primers (CTC-seq-F/R) for sequencing-based genotyping (Table S10).

### *SAG9* confers additive effects with ABA-related genes for rice storability and dormancy

**Figure 7.**
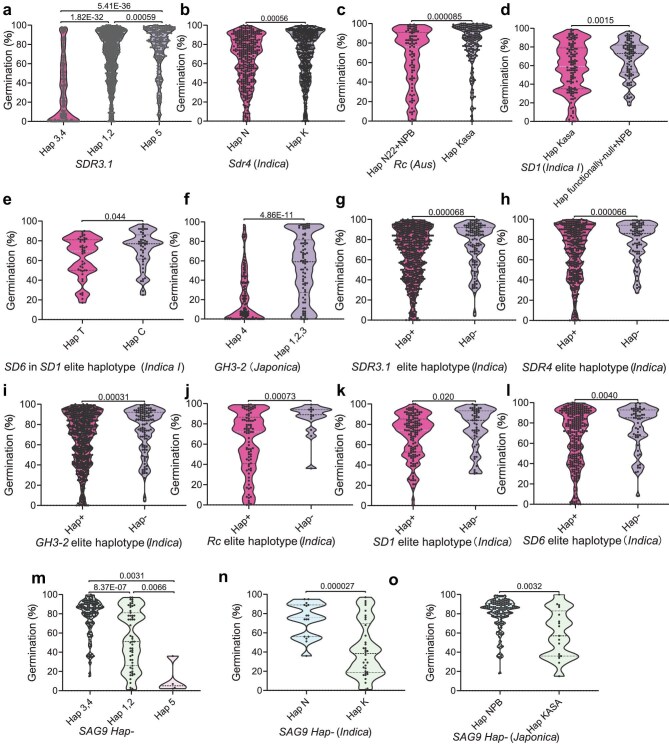
*SAG9* confers additive effects on rice seed storability and dormancy through genes related to ABA signaling. Germination of aged seeds with different haplotypes for *SDR3.1* (a), *Sdr4* (b), *Rc* (c)*, SD1* (d)*, SD6* (e), and *GH3-2* (f). Considering *Japonica* mainly harbors the *Sdr4* weak dormancy haplotype (Fig. S15l–n), *Indica* accessions were used in panel (b) to avoid interference of genetic heterogeneity, *Aus* accessions (*GH3-2, SAG9, SDR3.1*, and *SD1* were dominated by strong or weak haplotypes; Fig. S15p) were used in panel (c), and *Indica I* (*GH3-2, SAG9*, and *SDR3.1* were dominated by strong or weak haplotypes; Fig. S9e) accessions were used in panel (d). To eliminate the influence of *SD1* in *Indica I, SD1* elite haplotypes accessions of *Indica I* were used in panel (e). In view of *Indica* mainly harboring the *GH3-2* elite haplotype, *Japonica* accessions were used in panel (f). Germination of aged seeds between *SAG9* haplotypes *Hap+* and *Hap−* in backgrounds with elite haplotypes of *SDR3.1* (g), *Sdr4* (h), *GH3-2* (i), *Rc* (j), *SD1* (k), and *SD6* (l) in *Indica*. Two years of artificial-aging assays data obtained using 691 varieties were used in panels (a)–(l) as listed in Table S2m–o. Germination of freshly harvested seeds with different haplotypes of *SDR3.1* (m), *Sdr4* (n), and *Rc* (o) in the *SAG9* elite haplotype background. Published seed-dormancy data used for analyses in panels (m)–(o) are provided in Table S3. In view of *Japonica* mainly harboring a *Sdr4* weak dormancy haplotype (Fig. S15l–n), *Indica* accessions were used in panel (n). Considering only few *Indica* accessions harboring both *SAG9* and *Rc* elite haplotypes*, Japonica* accessions were used in panel (o). Dotted lines denote the 25th percentile, the median, and the 75th percentile. Statistical analysis was performed using one-way ANOVAs followed by Duncan’s new multiple range tests for panels (a) and (m) and two-tailed Student’s *t* tests for panels (b)–(l), (n), and (o); and numbers annotated for each comparison are the exact *P* values. ‘Elite haplotype’ refers to strong seed-storability or strong seed-dormancy haplotypes evaluated here.

Given that *SDR3.1, Sdr4, SD6*, and *Rc* regulate ABA biosynthesis or signaling in rice [15,33,34,59,60], we hypothesized that dormancy-associated genes may also regulate storability. Dormancy-enhancing haplotypes of *SDR3.1, Sdr4, SD6*, and *Rc* (but not *SD1*) increased seed storability (Fig. 7a and Fig. S15a–d). Since *Japonica* accessions predominantly carry dormancy-reducing haplotypes of these four genes and dormancy-enhancing haplotypes of *SD1* in *Japonica* (Fig. S16l–n), we conducted stratified analysis within the *Indica* subspecies to mitigate intersubpopulation confounding. This confirmed enhanced storability in *Sdr4* dormancy-enhancing haplotypes (Fig. 7b), whereas *SD1, Rc*, and *SD6* haplotypes showed statistically nonsignificant storability differences (Fig. S15e–g). Given that the effects of natural variation in *SD1, Rc*, and *SD6* are weak (Fig. S15j–l) and easily masked by genetic heterogeneity, we analysed refined genetic backgrounds: *Rc* haplotypes in *Aus* showed marked storability differences (Fig. 7c and Fig. S15h); *SD1* haplotypes in *Indica I* subgroup had differential storability (Fig. 7d and Fig. S15i); *SD6* haplotypes in the *SD1*-enhanced *Indica I* background displayed substantial variation (Fig. 7e). Additionally, the storability-enhancing haplotype of *GH3-2* increased longevity in *Japonica* (Fig. 7f), consistent with prior report [61]. Reverse-genetics validation demonstrated that *sdr3.1* mutants (*ko-1/ko-2*) enhanced dormancy [15] and storability versus ZH11 controls (Fig. S6g and h) and *sdr4* mutants (*cr-sdr4-1*/*cr-sdr4-2*) reduced dormancy [33] and storability versus N22 (Fig. S7c–f). These results establish that dormancy-related genes *SD1, SDR3.1, Sdr4, SD6*, and *Rc* regulate both seed dormancy and storability.

Additive genetic effects are known among *SD1, Sdr4*, and *Rc* in rice seed-dormancy regulation [33]. To characterize the additive interactions between *SAG9* and *SD1*/*SDR3.1*/*Sdr4*/*SD6*/*Rc*/*GH3-2*, we performed stratified analyses whereby accessions carrying storability-enhancing haplotypes for each gene were isolated within the *Indica* subspecies, followed by *SAG9* haplotype comparison. This revealed that *SAG9 Hap−* confers additional storability enhancement across all six gene backgrounds (Fig. 7g–l). Similarly, dormancy levels were further elevated in *SAG9 Hap−* accessions, when combined with *SDR3.1, Sdr4*, or *Rc* storability-enhancing haplotypes (Fig. 7m–o). Additive effects involving *SD1* and *SD6* were not analysed because of insufficient *Indica* accessions coharboring storability-enhancing haplotypes of both *SD1* and *SAG9*, and minimal intrinsic dormancy variation between *SD6* haplotypes (Fig. S15k). These results demonstrate that *SAG9* exhibits additive effects with *SD1, SDR3.1, Sdr4, SD6, Rc*, and *GH3-2* in coregulating seed storability and dormancy.

In addition, progressive pyramiding of storability-enhancing haplotypes (*SD1/SDR3.1/Sdr4/SD6/Rc/GH3-2/SAG9*) also enhanced seed storability and dormancy (Fig. S17a–d), indicative of additive effects between each other, which could be used for gene-pyramiding breeding. However, natural populations rarely accumulate all these optimal haplotypes (Table S7). Indeed, negative linkage was observed between *SAG9 Hap−* and other favorable alleles across varieties, with accessions bearing *SDR3.1/Sdr4/SD6/Rc/GH3-2* storability-enhancing haplotypes predominantly carrying *SAG9 Hap+* (Fig. S18a); 87.1% of *SAG9 Hap+* accessions harbor ≥2 storability-enhancing haplotypes of these five genes (Fig. S18b); and *SAG9 Hap+* frequency increased with the degree of pyramiding of *SDR3.1/Sdr4/SD6/Rc/GH3-2* (Fig. S18c). These findings further indicate the equalizer effect of *SAG9* to these five genes in seed dormancy and storability, which necessitate artificial intervention during breeding.

Moreover, the increasing average degree of pyramiding from the temperate *Japonica* (1.47) to the *Indica II* (4.45) subgroup implied their gradual increase in seed storability and dormancy (Fig. S18d). Varieties carried only one elite haplotype (*SD1/SDR3.1/Sdr4/SD6/Rc/GH3-2/SAG9*) mainly from *Temperate Japonica* and *Japonica Intermediate* subpopulations (Fig. S16l and m), which are predominantly distributed in high-latitude areas (Fig. S18d), indicating the adaptive advantage of weak dormancy (rapid germination) here. In contrast, varieties harboring six or seven elite alleles (*SD1/SDR3.1/Sdr4/SD6/Rc/GH3-2/SAG9*) are mostly from the *Indica II, Aus*, and *Indica Intermediate* subpopulations (Fig. S18d), which are mainly distributed in tropical regions (Fig. S16f, g, and p), and this reveals the adaptive advantage of germplasm with strong seed dormancy and storability in high-temperature and high-humidity environments. We have compiled the varieties with six or seven elite alleles to facilitate the selection of ideal breeding donors for breeders (Table S7).

### *SAG9* underwent balancing selection during rice domestication

**Figure 8.**
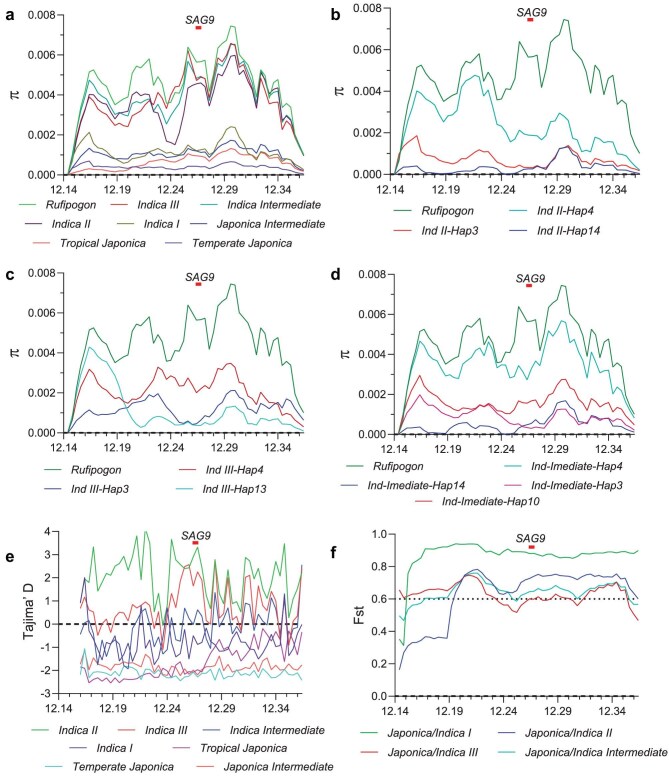
*SAG9* underwent balancing selection during rice domestication. (a) Nucleotide diversity of the *SAG9* locus in cultivated rice and wild rice populations. Nucleotide diversity of *SAG9* haplotypes in wild rice populations and *Indica II* (b), *Indica III* (c), and *Indica* Intermediate (d) subgroups. (e) Tajima’s D for the 0.2-Mb genomic region surrounding *SAG9* as determined using data from the 3000 Rice Genomes Project [54–56]. (f) *F*_ST_ values between *Indica* subgroups and *Japonica* in a 0.2-Mb genomic region surrounding *SAG9. Y* axis: average ThetaPi/site value in panels (a)–(e), average Tajima’s D value in panel (f), and weighted *F*_ST_ value in panel (c). *X*-axis values represent Nipponbare TIGR v7.0 genome coordinate on chromosome 9 (12.15–12.35 Mb) in which *SAG9* is indicated by red dashes. SNP data were obtained from the 446 *O. rufipogon* database [62] and 3000 Rice Genomes Project [54–56].

To elucidate *SAG9*’s domestication history, we calculated nucleotide diversity (π) and Tajima’ D across a 200-kb genomic region spanning *SAG9* in 446 wild-sequenced rice accessions [62] and the 3000 Rice Genomes Project [54–56]. Reduced π values were observed in cultivated subgroups: *Indica I, Tropical Japonica, Japonica Intermediate*, and *Temperate Japonica* subgroups as well as *SAG9* haplotypes within *Indica II, Indica III*, and *Indica Intermediate* subgroups (Fig. 8a–d), collectively indicating strong artificial selection. Tajima’s D values were all >0 in *Indica II, Indica III*, and *Indica Intermediate* subgroups (even exceeding 2.5 in *Indica II* and approaching 2 in *Indica III*, Fig. 8e). Correspondingly, both *Hap+* and *Hap−* were present in these three subgroups, and the nucleotide diversity corresponding to each haplotype was significantly lower than that in wild rice (Fig. 8b–d), indicating that both haplotypes have undergone selection here. In addition, both haplotypes (*Hap+* and *Hap−*) have been stably maintained in nature over long periods (Fig. S10i and Table S4). Functional *SAG9* (*Hap+*) reduces seed dormancy and storability while enhancing seed vigor in the early storage stage, mediating a trade-off between early vigor and dormancy/storability, and is predominantly distributed in *Indica* subpopulation (Fig. S10e). By contrast, *Hap−* is predominantly distributed in *Japonica* subpopulation (Fig. S10e). Within *Indica, Hap−* is restricted to tropical regions (Fig. S10h), whereas *Hap+* in *Japonica* is concentrated in high-latitude areas (Fig. S10g). Collectively, these results demonstrate that *SAG9* was subjected to environmentally heterogeneous balancing selection during rice domestication.

Subspecies-specific selection shaped *SAG9* haplotype distribution. Integration with haplotype-frequency data (Fig. S10a) revealed distinct selection patterns: the *Indica I* subgroup mainly selected high-vigor haplotypes *Hap+* (*Hap3* and *Hap10*), with *Tropical Japonica* and *Japonica Intermediate* subgroups having mainly selected the strong-storability/dormancy haplotype *Hap−* (*Hap11*), and both functional haplotypes were selected in *Indica II* (*Hap+*: *Hap3* and *Hap4; Hap−*: *Hap14*), *Indica III* (*Hap+*: *Hap3* and *Hap4; Hap−*: *Hap13*), *Indica* intermediate (*Hap+*: *Hap3, Hap4*, and *Hap10; Hap−*: *Hap14*), and *Temperate Japonica (Hap+*: *Hap1; Hap−*: *Hap16*) subgroups. Consistent with this, strong divergent selection was evidenced by exceptionally high *F*_st_ (fixation index, *F*_st_ > 0.6) values at the *SAG9* locus (Fig. 7f), aligning with subspecies-specific haplotype preferences.

## DISCUSSION

### *SAG9* is a promising target for rice breeding to achieve ideal dormancy and strong storability

Seed storability and dormancy represent complex domestication traits governed by polygenic networks and genotype*–*environment interactions [3,5,6]. Despite their agricultural significance, their molecular mechanisms remain largely elusive, with few causal genes functionally validated. Our study establishes *SAG9* as the causal gene for the major QTL *qSS-9*, explaining 49.9%*–*52.9% of phenotypic variance in seed storability across environments [35,36]. *SAG9* simultaneously modulates dormancy. The elite haplotype *Hap−* greatly enhances seed storability while maintaining moderate dormancy levels. Knockout of *SAG9* in modern varieties endows seeds with improved storability and dormancy without compromising major agronomic traits. These findings position *SAG9* as an ideal target for molecular breeding of dual-trait enhancement.

### The *SAG9* pathway coordinately regulates seed dormancy and storability

*bZIP10/23/66/72* encode bZIP-type transcription factors serving as positive regulators of rice seed dormancy [15,16,50,63]. *EM1, LEA3*, and *Rab16A* encode LEA proteins, previously established as ABA signaling markers that positively regulate seed dormancy [15,16]. This study demonstrates that bZIP10/23/46/66/72 additionally enhance seed storability by directly binding to promoters of three *LEA* genes (*EM1, LEA3*, and *Rab16A*) to activate their expression (Fig. S19). LEA proteins confer desiccation tolerance through the formation of glassy cytoplasm [6,17], which can restrict molecular mobility, reduce cellular metabolic activity, and stabilize cellular components—mechanisms that suppress seed aging and dormancy release [5,22,24,64]. SAG9 suppresses this bZIP-mediated transcriptional activation, thereby negatively regulating both traits in Nipponbare (Fig. S19a). Conversely, in Kasalath, altered subcellular localization and disrupted protein interactions of SAG9^Kas^ weaken this suppression (Fig. S19b). This work establishes the SAG9–bZIPs–LEAs pathway as a molecular link between dormancy and storability. Consequently, selecting for enhanced seed-dormancy genotypes may concurrently improve seed storability, enabling streamlined breeding programs targeting these coupled traits. Collectively, our work reveals that *SAG9* represses the downstream transduction of ABA signaling. However, its regulatory relationships with other ABA pathway components (*PYLs, PP2Cs*, or *SnRKs*) remain unclear, which warrants further investigation.

*SAG9* mRNA accumulates predominantly, implying its important role in this tissue. Previously reported rice genes exhibiting a similar expression pattern primarily comprise *OsGF14h* (involved in temperature-dependent and submergence-tolerant germination) [65,66] and endosperm storage substances (LEAs, starch, and protein) synthesis-related genes [67–70]. These findings indicate that *SAG9* may be associated with the accumulation of storage substances in the endosperm, and some of these substances (including LEAs) might be involved in the establishment of seed dormancy and storability. However, the identification of other key substances (excluding LEAs) in this process warrants further investigation. In addition, previous studies have demonstrated that the maintenance of endosperm quality is critical for seed storability and germination in *A. thaliana* [71]. Whether *SAG9* preferentially induces endosperm cell death during storage, thereby leading to germination failure requires further research.

### *SAG9* serves as an equalizer and major target in the domestication of seed vigor

Seed vigor represents a direct target of selective stress during rice domestication. Elite *SD1, SDR3.1, Sdr4, SD6, Rc*, and *SAG9* haplotypes that enhance seed storability prolong vigor retention under prolonged storage but concurrently delay vigor recovery through reinforced seed dormancy during after-ripening. Domestication-driven selection for strong vigor has favored weak dormancy alleles across multiple loci [15,33,34], particularly in high-latitude regions. Conversely, in tropical regions, seeds require strong storability to maintain vigor under conditions of elevated temperature and humidity. This geographic variation in adaptive advantages has driven selection for different alleles across regions, i.e. balancing selection.

Functional *SD1, SDR3.1, SD6, GH3-2*, and *SAG9* are negative regulators of seed dormancy and storability, while functional *Sdr4* and *Rc* are positive regulators. Based on the geographical distribution of their haplotypes (Fig. S16), we summarized the domestication trajectory of these regulators. In wild rice, elite haplotypes of *Sdr4, SDR3.1, SD6, GH3-2*, and *Rc* had evolved for strong dormancy and storability, while functional *SD1* and *SAG9* had evolved to ensure rapid dormancy breakage and timely germination (Fig. S20a). Similarly, in initial domesticated *Indica*, accessions with conserved elite haplotypes of *SDR3.1* and *GH3-2* maintain robust baseline dormancy and storability. Concurrently, *SAG9 Hap+* was selected to counterbalance excessive dormancy (Fig. S20b). In contrast, in initial domesticated *Japonica*, nearly all accessions carry weak-dormancy/storability haplotypes at *SDR3.1, Sdr4, SD6*, and *Rc* loci, resulting in reduced storability. This deficit drives the retention of *SAG9 Hap−* to enhance seed storability (Fig. S20c). As cultivation extended into tropical regions, intensified selection for storability under high temperature/humidity promoted stronger fixation of elite haplotypes of *SAG9* and *Sdr4* in *Indica* (Fig. S20d) as well as elite haplotypes of *SAG9* and *GH3-2* in *Japonica* (Fig. S20e). As cultivation extended into high-latitude regions, intensified selective stress for faster germination promotes greater selection of *SAG9 Hap+* to accelerate dormancy release (Fig. S20f).

This model establishes *SAG9* as a bidirectional adaptive switch, with *Hap+* promotes dormancy breakage for high-latitude adaptation, while *Hap−* boosts storability under tropical conditions. The combinatorial diversity of haplotypes across *SD1, SDR3.1, Sdr4, SD6, Rc, GH3-2*, and *SAG9* reflects adaptive optimization of rice seeds to environmental constraints and human selection pressures. Among them, *SAG9* acts as a genetic equalizer and major target in the domestication of seed dormancy or storability: *SAG9 Hap+* counteracts excessive dormancy in *Indica*, while *SAG9 Hap−* compensates for weak storability in *Japonica*, reflecting its antagonistic effects with *GH3-2/SDR3.1/Sdr4/SD6/Rc*; the combinations of *SAG9 Hap−* with the elite haplotypes of *Sdr4* or *GH3-2* in tropical regions reflect its synergistic effects. Whether these genes have genetic interactions with *SAG9* remains unclear, and this will be one of the key focuses of our follow research.

Notably, OsGF14h positively regulates submergence tolerance and temperature-dependent germination by interacting with ABI5, VP1, and HOX3 [65,66]. It represses the expression of the ABA receptor gene *PYL5* and ABA signaling marker genes (*EM1, LEA3*, and *Rab16A*), while promoting the expression of *OsGA20ox1*, a key gene involved in GA biosynthesis [65,66]. This molecular mechanism is similar to that of *SAG9*. In addition, the nonfunctional allele of *OsGF14h* was selected during rice domestication to counterbalance the reduced seed dormancy caused by *Rc* and *Sdr4* alleles [65,66], which also resembles the domestication model of *SAG9*. Whether *OsGF14h* genetically interacts with *SAG9* and regulates seed storability warrant further investigation.

Additive genetic effects among *SD1, SDR3.1, Sdr4, SD6, Rc*, and *SAG9* indicate that pyramiding elite alleles at these loci provides an effective strategy to enhance seed dormancy and storability. Furthermore, we have identified varieties that have already pyramided six or seven elite haplotypes of these genes (Table S9), which serve as optimal donor germplasm for breeding programs. To resolve the trade-off between seed storability and the performance of vigor, we propose a precision breeding framework for ideal seeds: stage-inducible expression of negative regulators (*SAG9, SD1, SDR3.1, SD6*, and *GH3-2*) during after-ripening to accelerate dormancy release; with concomitant induction of positive regulators (*Rc* and *Sdr4*) during preharvest and storage phases to bolster vigor and storability. This spatiotemporal regulation strategy is expected to synchronously optimize all three traits (dormancy, vigor, and storability). However, the identification and validation of seed-specific promoters compatible with such temporal control, along with practical implementation feasibility, is first required.

## MATERIALS AND METHODS

The methods and materials are described in detail in the Supplementary data.

## Supplementary Material

nwag248_Supplemental_File

## Data Availability

The sequence data from this study can be found at Rice Genome Annotation Project website (http://rice.uga.edu/) under the following accession numbers: *SAG9* (*LOC_Os09g20400* [https://rice.uga.edu/cgi-bin/ORF_infopage.cgi?orf=LOC_Os09g20400]), *bZIP10/ABI5* (*LOC_Os01g64000* [http://rice.uga.edu/cgi-bin/ORF_infopage.cgi? orf = LOC_Os01g64000]), *bZIP23* (*LOC_Os02g52780* [https://rice.uga.edu/cgi-bin/ORF_infopage.cgi?orf=LOC_Os02g52780]), *bZIP40* (*LOC_Os05g36160* [https://rice.uga.edu/cgi-bin/ORF_infopage.cgi?orf=LOC_Os05g36160]), *bZIP46* (*LOC_Os06g10880* [https://rice.uga.edu/cgi-bin/ORF_infopage.cgi?orf=LOC_Os06g10880]), *bZIP66/TRAB1* (*LOC_Os08g36790* [http://rice.uga.edu/cgi-bin/ORF_infopage.cgi? orf = LOC_Os08g36790]), *bZIP72* (*LOC_Os09g28310* [http://rice.uga.edu/cgi-bin/ORF_infopage.cgi?orf=LOC_Os09g28310]), *PER1A* (*LOC_Os07g44430* [https://rice.uga.edu/cgi-bin/ORF_infopage.cgi?orf=LOC_Os07g44430]), *EM1* (*LOC_Os05g28210* [http://rice.uga.edu/cgi-bin/ORF_infopage.cgi? orf = LOC_Os05g28210]), *LEA3* (*LOC_Os05g46480* [https://rice.uga.edu/cgi-bin/ORF_infopage.cgi?orf=LOC_Os05g46480  *Rab16A* (*LOC_Os11g26790* [https://rice.uga.edu/cgi-bin/ORF_infopage.cgi?orf=LOC_Os11g26790  *SD1* (*LOC_Os01g66100* [https://rice.uga.edu/cgi-bin/ORF_infopage.cgi?orf=LOC_Os01g66100]), *SDR3.1/MODD* (*LOC_Os03g11550* [https://rice.uga.edu/cgi-bin/ORF_infopage.cgi?orf=LOC_Os03g11550  *Sdr4* (*LOC_Os07g39700* [https://rice.uga.edu/cgi-bin/ORF_infopage.cgi?orf=LOC_Os07g39700]), *SD6* (*LOC_Os06g06900* [https://rice.uga.edu/cgi-bin/ORF_infopage.cgi?orf=LOC_Os06g06900]), *Rc* (*LOC_Os07g11020* [https://rice.uga.edu/cgi-bin/ORF_infopage.cgi?orf=LOC_Os07g11020]), and *GH3-2* (*LOC_Os01g55940* [https://rice.uga.edu/cgi-bin/ORF_infopage.cgi?orf=LOC_Os01g55940]). Source data are provided with this paper.
