# Supplementary material for: Superior alleles of SEED-AGING GENE 9 enhance seed storability and dormancy in rice
Source: Natl Sci Rev. 2026 Apr 29;13(10):nwag248. doi: 10.1093/nsr/nwag248 (PMC13248866; doi:10.1093/nsr/nwag248)
Supplement: nwag248_Supplemental_File [file nwag248_supplemental_file.docx]

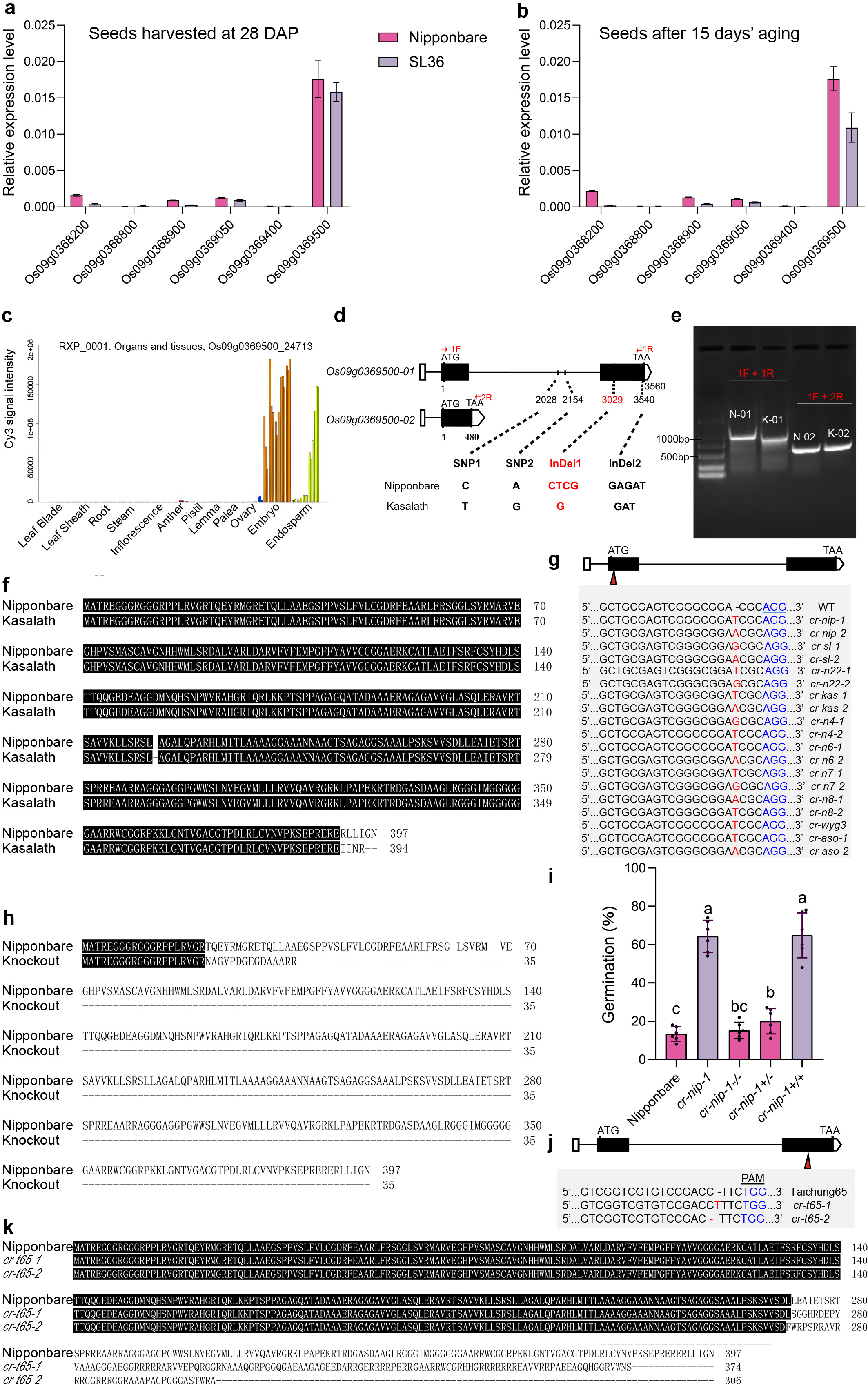


**Supplementary Fig.1** **Identification of the *qSS-9* locus in rice.**

**a**,**b**, Expression of six candidate genes in Nipponbare and SL36 at seed-maturation stage (**a**) and after accelerated aging (**b**). DAP, days after pollination. Data are presented as mean ± s.d of n = 3 replicates. **c**, Organ-level expression data for *Os09g0369500* obtained from the Rice Expression Profile Database (RiceXPro: ricexpro.dna.affrc.go.jp). **d**, Structure of *Os09g0369500* based on genomic sequencing. *Os09g0369500-01* and *Os09g0369500-02* represent its two transcripts. Black lines represent introns, white boxes represent non-coding regions, black boxes represent coding regions. Red arrows indicate the positions of primers used for PCR identification. **e**, Agarose gel electrophoresis image of transcripts *Os09g0369500-01* and *Os09g0369500-02*. cDNAs from mature seeds of Nipponbare and Kasalath were used as templates, respectively. The positions of specific primers used for amplification are marked with red arrows in (**d**), and the specific sequences are provided in Supplementary Table **10**. **f**, Alignment of Os09g0369500 protein sequences from Nipponbare and Kasalath. Black shading represents conserved residues. **g**, Location and genotypes of sites mutated in *Os09g0369500* using CRISPR–Cas9 gene editing in various wild-type backgrounds. The red arrow indicates the site targeted for mutagenesis in exon 1. Red font indicates mutation sites, blue font indicates the position of the protospacer adjacent motif (PAM) sequence. **h**, Alignment of protein sequence of *Os09g0369500* in Nipponbare and knockout lines in various wild-type backgrounds. Black shading represents conserved residues. All mutant alleles generated in different backgrounds encoded a similar, truncated peptide. **i**, Germination of Nipponbare, *cr-nip-1* and heterozygous seeds. Data are presented as mean ± s.d of n = 6 replicates. Heterozygous seeds were obtained from a cross between Nipponbare and *cr-nip-1*. *cr-nip+/-*, heterozygous in *Os09g0369500*; *cr-nip+/+*, homozygous in *Os09g0369500* as *cr-nip-1*; *cr-nip-/-*, homozygous in *Os09g0369500* as Nipponbare. Statistical analysis was performed using one-way ANOVA followed Duncan’s new multiple range test; different letters indicate a statistically significant difference at *P* < 0.05. **j**, Location and genotypes of sites mutated in *Os09g0369500* using CRISPR–Cas9 gene editing in cv. Taichung65. The red arrow indicates the site targeted for mutagenesis in exon 1. Red font indicates mutation sites, blue font indicates the position of the protospacer adjacent motif (PAM) sequence. **k**, Protein sequence of *Os09g0369500* in Taichung65 and knockout lines generated in this background. Black shading represents conserved residues.


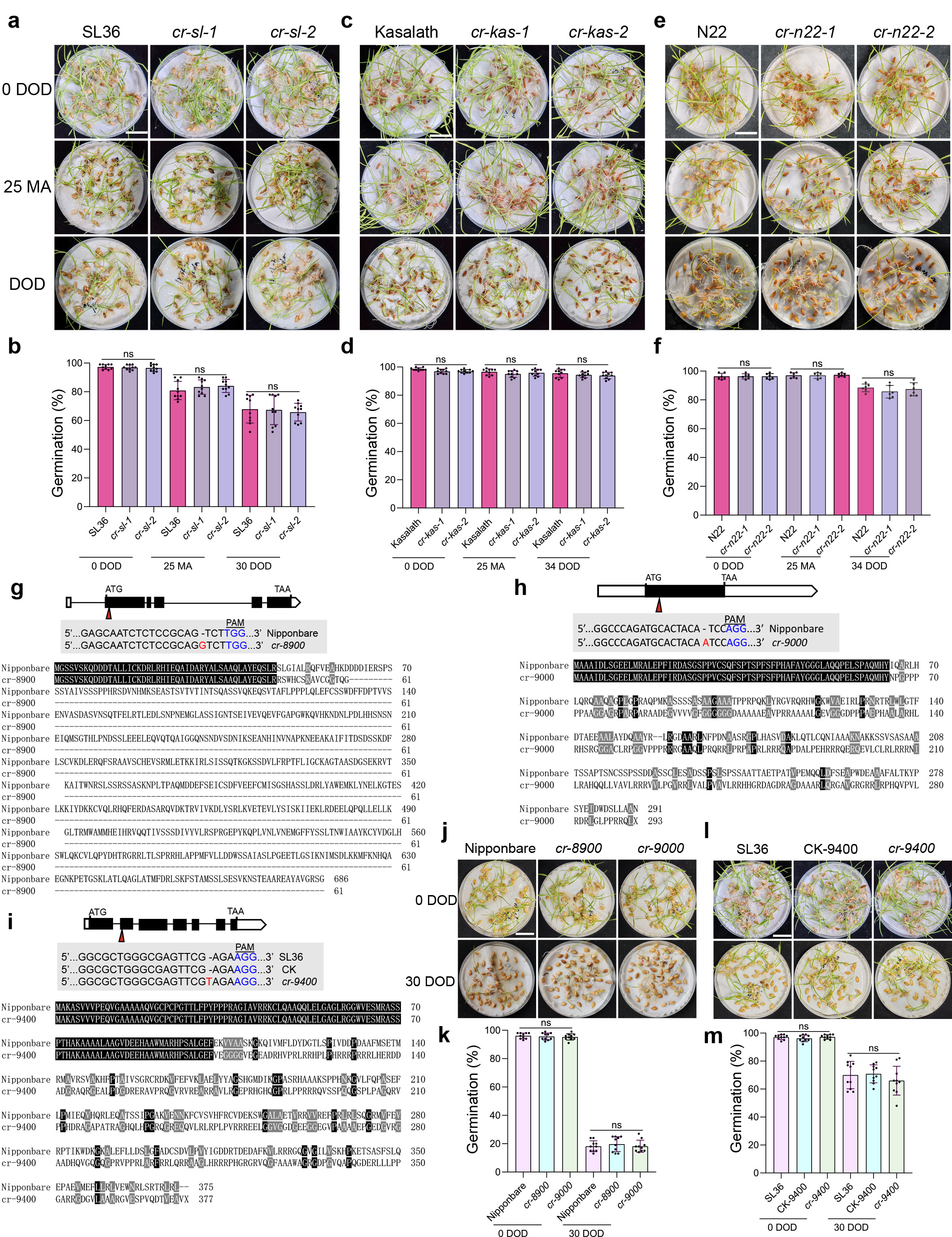


**Supplementary Fig. 2 *Os09g0369500* is the causal gene underlying *qSS-9* locus**.

**a**,**c**,**e**, Germination performance for SL36 (**a**), Kasalath (**c**) and N22 (**e**) wild types and respective pairs of mutant alleles. Scale bars: 2 cm. The *Os09g0369500* sequence in N22 is identical to Kasalath. Duration of artificial aging was 30 d in SL36 (**a**), 34 d in Kasalath (**c**) and 34 d in N22 (**e**) background. **b**,**d**,**f**, Germination quantification of SL36 (**b**), Kasalath (**d**) and N22 (**f**) wild types and respective pairs of mutant alleles. Data are presented as mean ± s.d of n = 10 for SL36 and Kasalath and respective mutants, n = 6 for N22 and its mutants. **g–i**, Mutation sites and respective protein sequence aligning in *Os09g0368900* (**g**), *Os09g0369000* (**h**) and *Os09g0369400* (**i**) alleles obtained by gene editing in the Nipponbare or SL36 background. Red arrows indicate sites targeted for mutagenesis. Red font indicates mutation sites and blue font indicates the position of the protospacer adjacent motif (PAM) sequence. Black shading represents conserved residues. **j**,**l**, Germination performance for Nipponbare and mutant alleles of *Os09g0368900* and *Os09g0369000* (**j**) and SL36, CK-9400 and *Os09g0369400* mutant allele (**l**), CK-9400 indicates the unedited negative control line. Scale bars: 2 cm. **k**,**m**, Germination quantification for Nipponbare and mutants in *Os09g0368900* and *Os09g0369000* (**k**), and SL36 and mutants in *Os09g0369400* (**m**). Data are presented as mean ± s.d of n = 10. DOD, days of deterioration; MA, months of natural aging. Photographs were taken 7–10 d after imbibition. Statistical analysis was performed using one-way ANOVA followed Duncan’s new multiple range tests for mutants versus respective wild types within each time point; ns, non-significant difference.


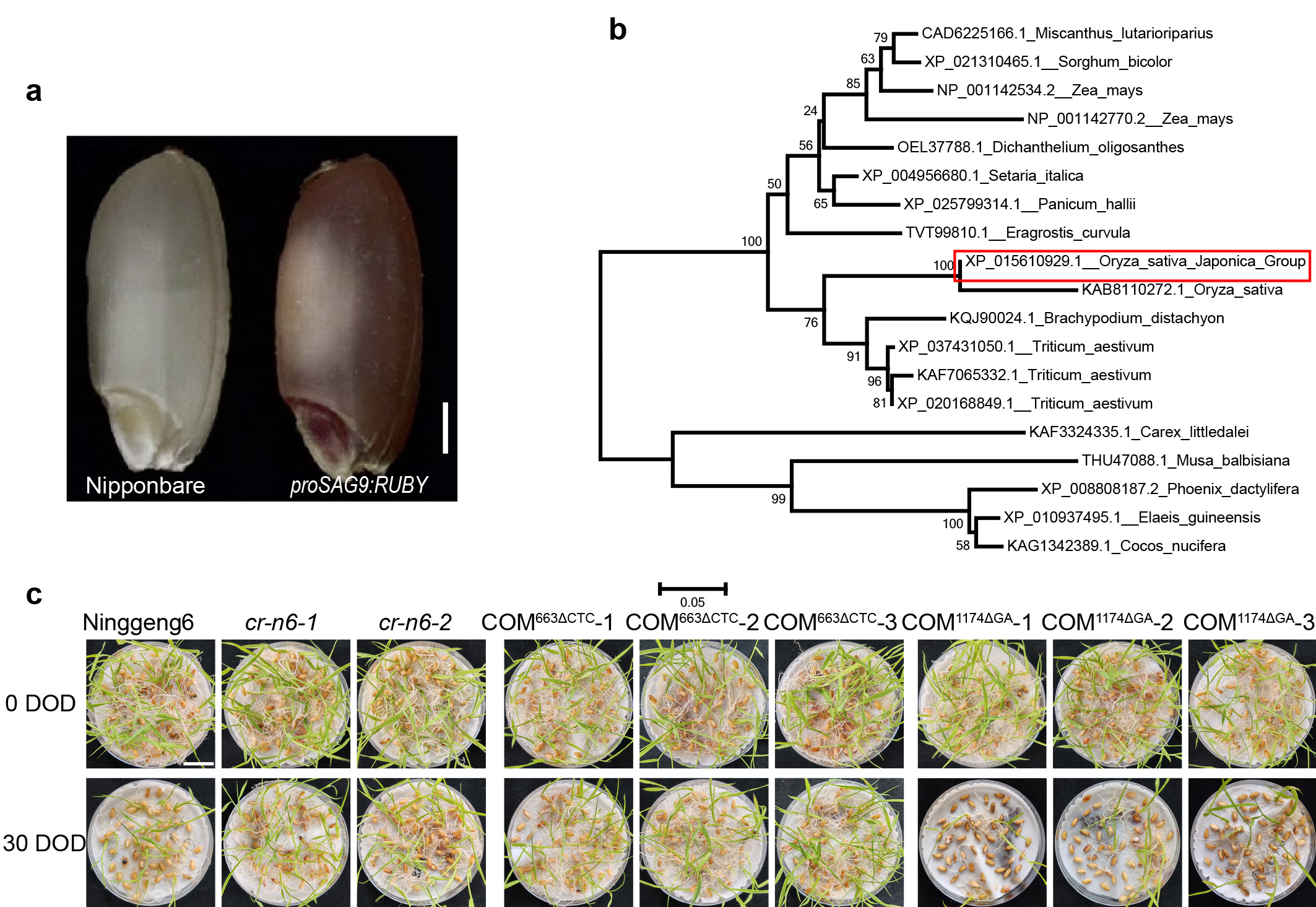


S**upplementary Fig. 3 SAG9 is a nuclear-localized protein lacking known domains.**

**a**, Representative wild-type cv. Nipponbare and transgenic *proSAG9:RUBY* seeds in the Nipponbare background harvested at 35 DAP. A 2.4-kb *SAG9* promoter was cloned from Nipponbare and fused to *RUBY* [1]. DAP, days after pollination. Scale bar = 1 mm. **b**, Phylogenetic analysis of SAG9. Bootstrap values for which the associated taxa clustered together are shown next to the branches. The tree is drawn to scale, with branch lengths measured in the number of substitutions per site (bar: 0.05). The analysis used 22 sequences and SAG9 from *Japonica* is marked in a red box. **c**, Germination performance wild-type cv. Ninggeng6, *sag9* mutant alleles in this background (*cr-n6-1/-2*) and various transgenic *cr-n6-2* complementation lines. COM^663ΔCTC^: complemented with native-promoter-driven *SAG9^c.663–665ΔCTC^*; COM^1174ΔGA^, complemented with native-promoter-driven *SAG9^c.1174_1175ΔGA^*. Scale bar: 2 cm.


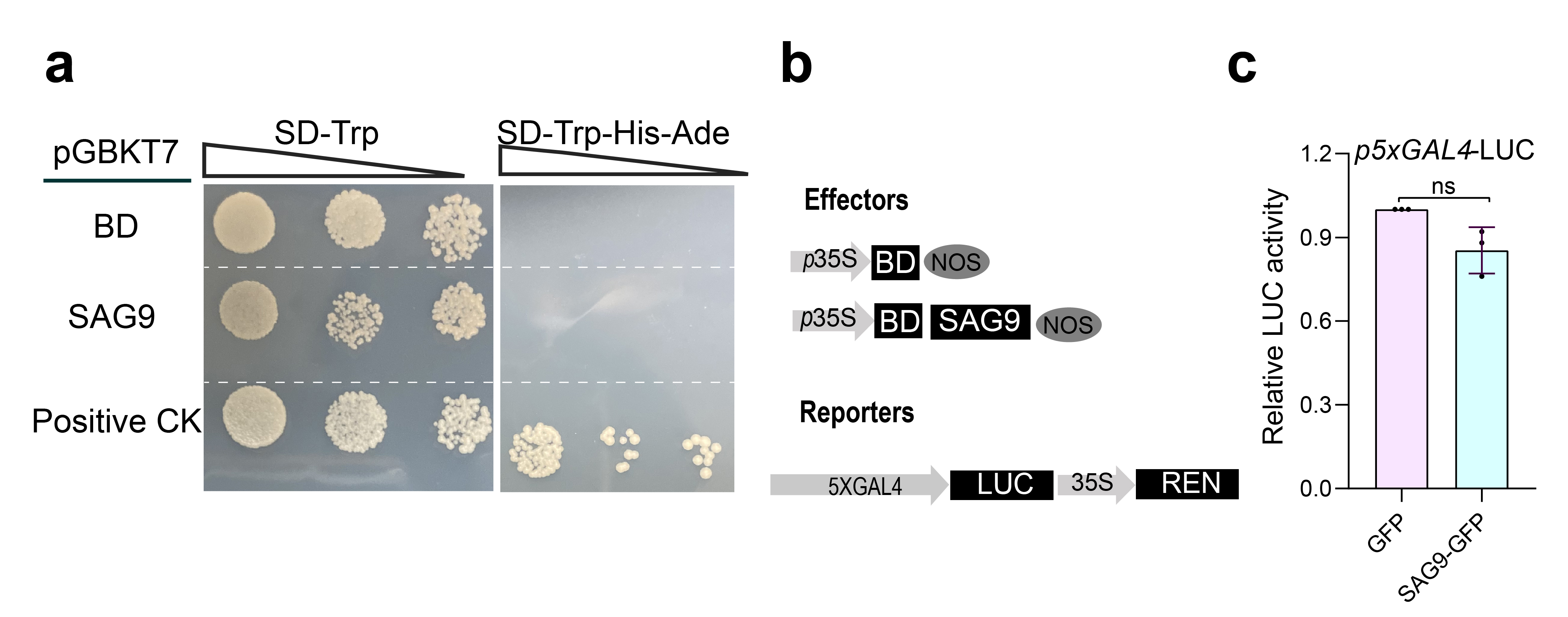


S**upplementary Fig. 4 SAG9 lacks detectable transcriptional-activation activity.**

**a**, SAG9 transcriptional-activation activity test in yeast. **b**, Effector and reporter plasmids used in transient dual-luciferase reporter assays in rice protoplasts in (**c**). **c**, Dual-luciferase reporter assay in rice protoplasts. N = 3, each biological replicates was comprised independent technical replicates. Data are presented as mean ± s.d of n = 3 replicates. Statistical analysis was performed using two-tailed Student's *t* test; ns, non-significant difference.


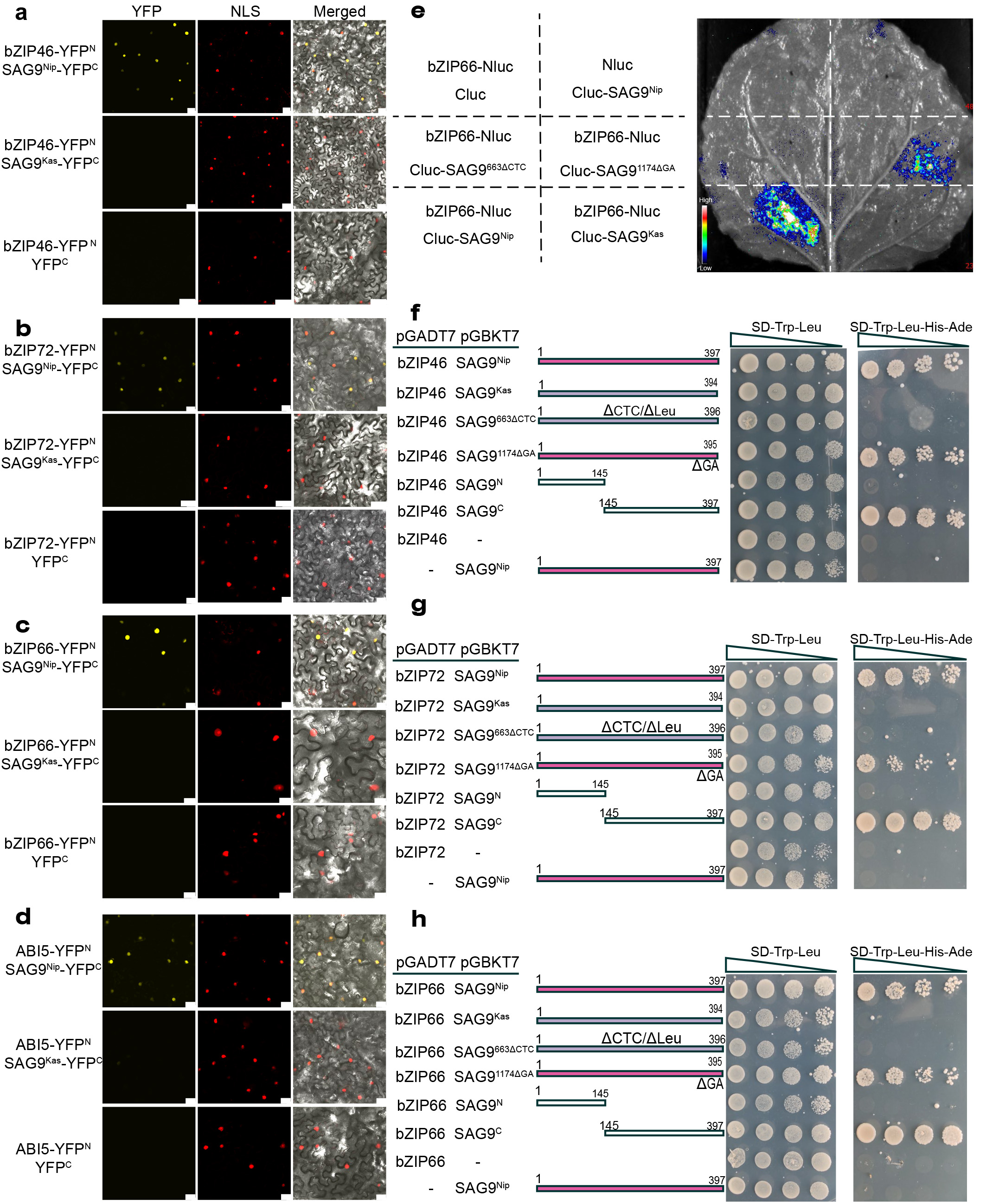


S**upplementary Fig. 5 The SAG9 C-terminus and a CTC triplet are required for its physical interaction with bZIP transcription factors.**

**a–d,** Bimolecular fluorescence-complementation assays of the physical interaction between SAG9 and bZIP46 (**a**), bZIP72 (**b**), bZIP66 (**c**) and bZIP10/ABI5 (**d**). **e**, Luciferase-complementation imaging assay of the physical interaction between SAG9 and bZIP66. Scale bars: 10 μm. **f–h**, Yeast two-hybrid assays of the physical interaction between SAG9 and bZIP46 (**f**), bZIP72 (**g**) and bZIP66 (**h**). SAG9^Nip^: cloned from Nipponbare, SAG9^Kas^: cloned from Kasalath, SAG9^663ΔCTC^: SAG9^Nip^ with a CTC deletion from position +663, SAG9^1174ΔGA^: SAG9^Nip^ with a GA deletion from position +1,174; SAG9^N^: amino acids 1–145 of SAG9^Nip^; SAG9^C^: amino acids 145–397 of SAG9^Nip^.


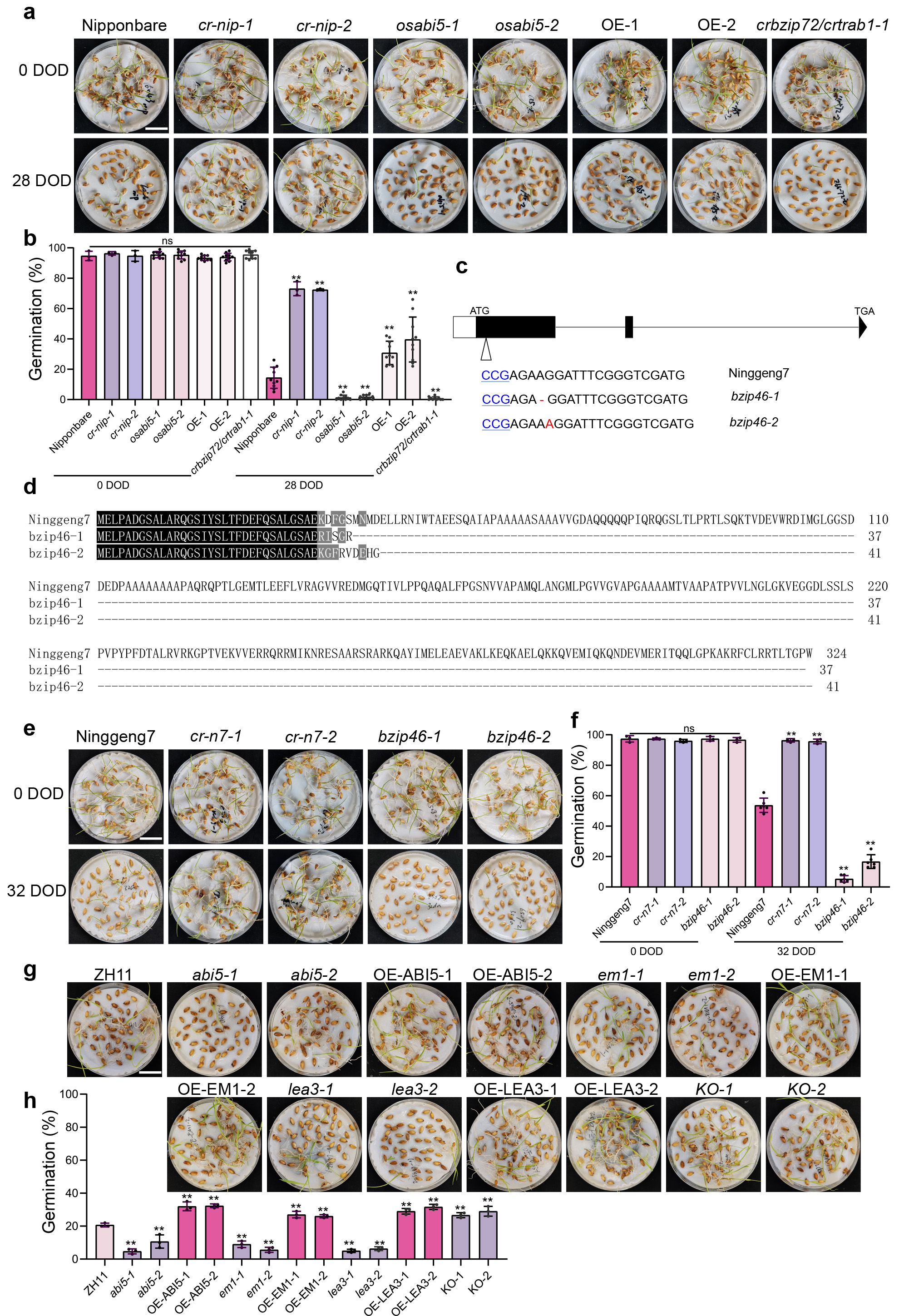


**Supplementary Fig. 6 Select rice *bZIP* genes positively regulate seed storability.**

**a**,**b**, Germination performance (**a**) and quantification (**b)** of wild-type cv. Nipponbare, *bzip10/abi5*, *bzip66/trab1* and *bzip72* mutants, and transgenic *ABI5* over-expression lines in the Nipponbare background. N = 3 for 0 DOD Nipponbare, *cr-nip-1* and *cr-nip-2;* n = 10 for all other genotypes. Each biological replicate comprised three independent technical replicates. Scale bar: 2 cm. **c**, Mutant sites in *bzip46* mutant alleles developed by gene editing in the indicated backgrounds. Thin black lines represent introns, white boxes represent non-coding regions, black boxes represent coding regions, arrows indicate sgRNA binding sites, red font indicates detected mutations and blue font indicate the position of the protospacer adjacent motif (PAM). **d**, Alignment of protein sequence of *bZIP46* in Ninggeng7 and knockout lines. Black shading represents conserved residues. All mutant alleles encoded truncated peptide. **e**,**f**, Germination performance (**e**) and quantification (**f**) of wild-type cv. Ninggeng7, *sag9* mutants and *bzip46* mutants seeds. N = 3 for 0 DOD and n = 6 for 32 DOD, each biological replicate comprised three independent technical replicates. Scale bars: 2 cm. **g**,**h**, Germination performance (**g**) and quantification (**h**) of wild-type cv. ZH11, *abi5-1*, *abi5-2* mutants, transgenic *bZIP10/ABI5* over-expression lines (OE-ABI5-1, OE-ABI5-2), *em1-1*, *em1-2* mutants, transgenic *EM1* over-expression lines (OE-EM1-1, OE-EM1-2), *lea3-1*, *lea3-2* mutants, transgenic *LEA3* over-expression lines (OE-LEA3-1, OE-LEA3-2), and *sdr3.1* mutants (*KO-1*, *KO-2*) after 24 days’ artificial-aging. N = 3, each biological replicate comprised three independent technical replicates. Scale bars: 2 cm. DOD, days of deterioration. Photographs were taken 7–10 d after imbibition; Data are presented as mean ± s.d. Statistical analysis was performed using one-way ANOVA followed Duncan’s new multiple range tests versus respective wild types within each time point; ns, non-significant difference; singular asterisk and different letters indicate a statistically significant difference at *P* < 0.05; two asterisks, *P* < 0.01.


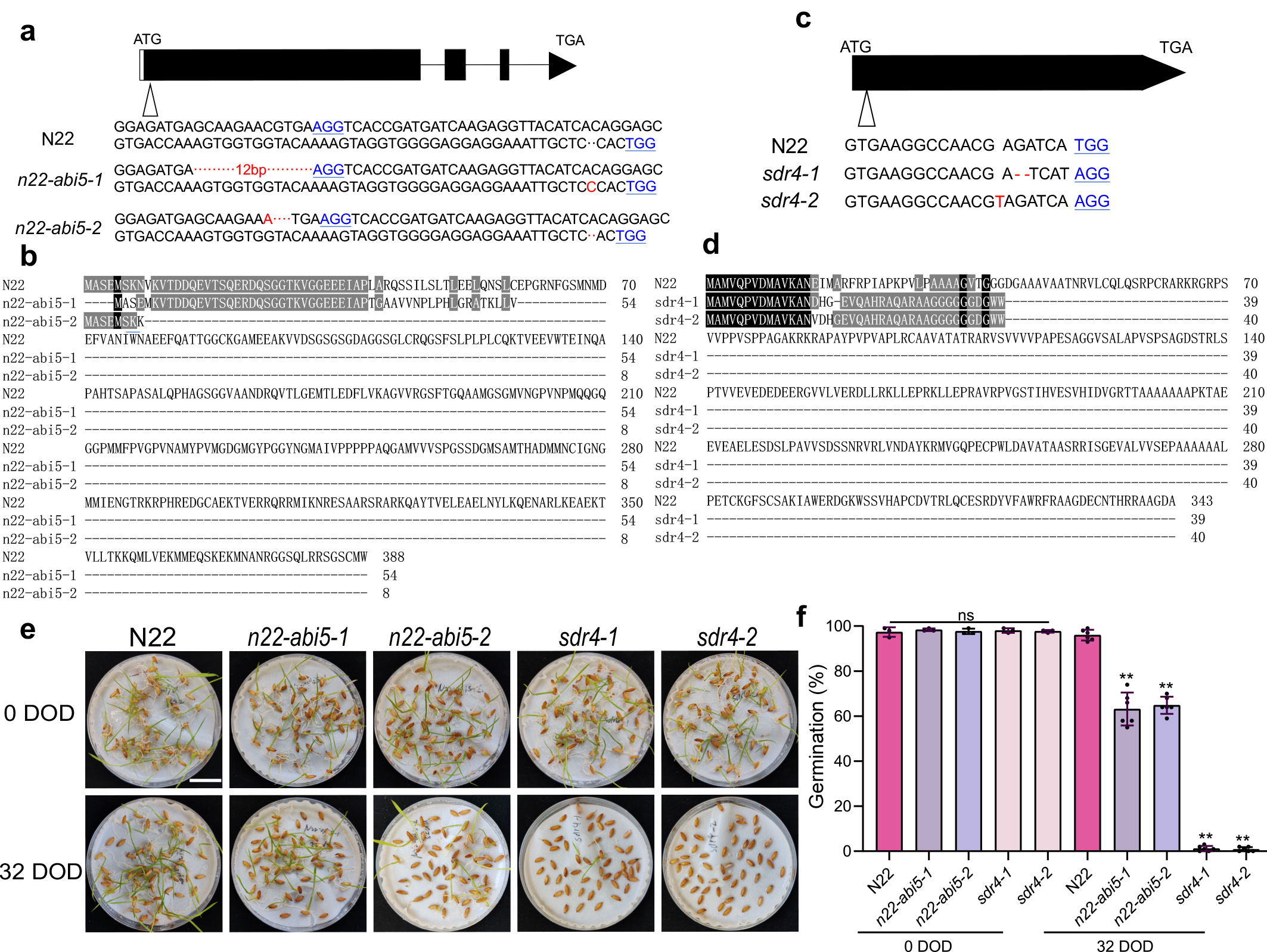


**Supplementary Fig. 7 Rice *bZIP10*/*ABI5* and *Sdr4* positively regulate seed storability.**

**a**,**c**, Mutant sites in *bzip10/abi5* (**a**) and *sdr4* (**c**) mutant alleles developed by gene editing in N22 background. Thin black lines represent introns, white boxes represent non-coding regions, black boxes represent coding regions, arrows indicate sgRNA binding sites, red font indicates detected mutations and blue font indicate the position of the protospacer adjacent motif (PAM). **b**,**d**, Alignment of protein sequence of *bzip10/abi5* (**b**) and *sdr4* (**d**) in N22 and respective knockout lines. Black shading represents conserved residues. All mutant alleles encoded truncated peptide. **e**,**f**, Germination performance (**e**) and quantification (**f**) of wild-type cv. N22, *bzip10/abi5* mutants and *sdr4* mutants seeds before or after aging. N = 3 for 0 DOD and n = 6 for 32 DOD. Each individual replicate comprised three independent technical replicates. Photographs were taken 7–10 d after imbibition. Scale bars: 2 cm. Data are presented as mean ± s.d. Statistical analysis was performed using one-way ANOVA followed Duncan’s new multiple range tests versus respective wild types within each time point; ns, non-significant difference; singular asterisks and different letters indicate a statistically significant difference at *P* < 0.05; two asterisks, *P* < 0.01.


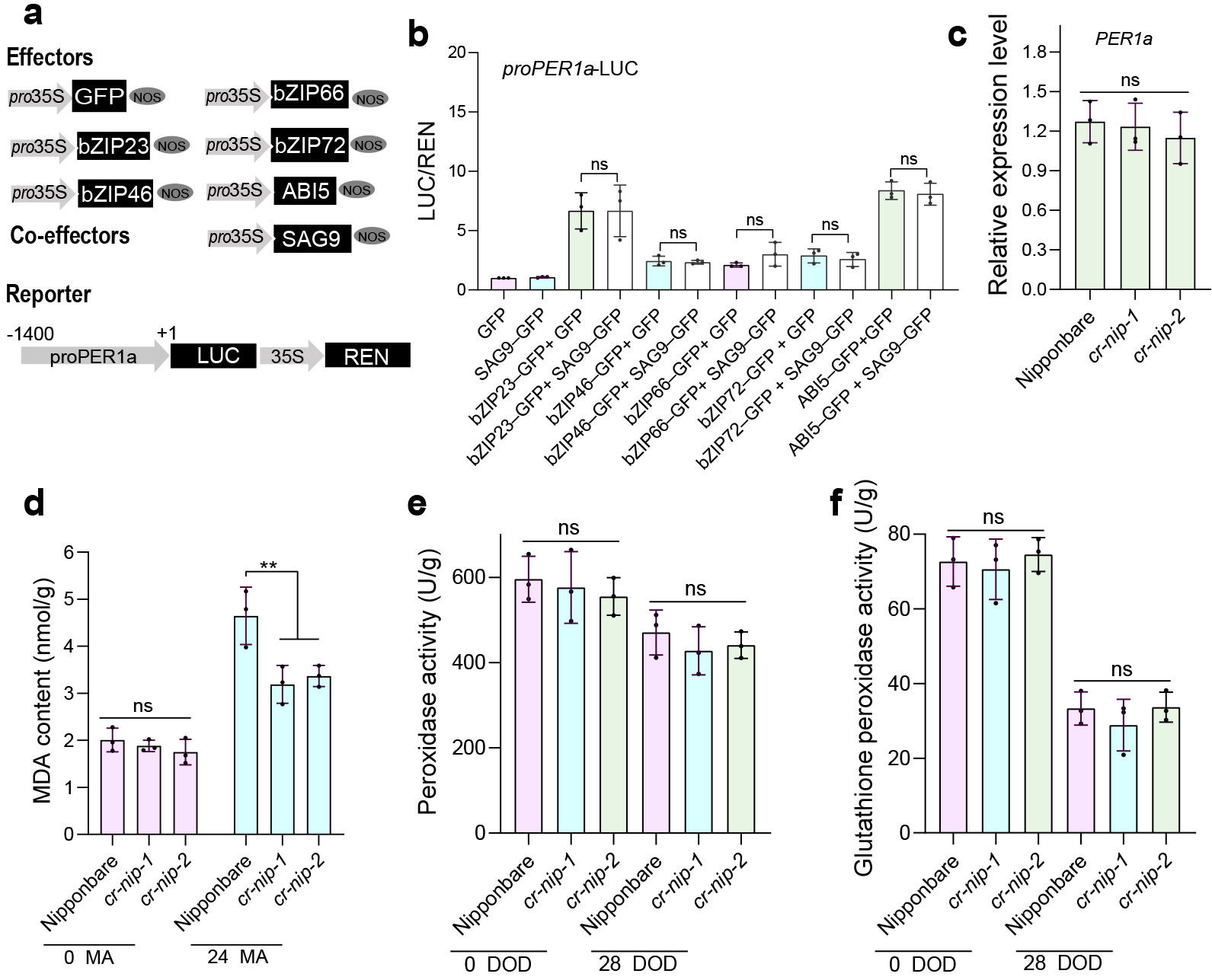


**Supplementary Fig. 8 SAG9 does not obviously influence the expression of *PER1A*.**

**a**, Effector and reporter plasmids used in transient dual-luciferase reporter assays in rice protoplasts. **b**, LUC/REN values reporting *PER1A* promoter activity*.* Statistical analysis was performed using one-way ANOVA followed Duncan’s new multiple range tests versus respective effector without co-transform of SAG9-GFP; ns, non-significant difference. **c**, *PER1A* expression levels in Nipponbare and two *sag9* mutant seeds harvested at 35 DAP. DAP, days after pollination. **d–f**, MDA content (**d**), peroxidase activity (**e**) and glutathione peroxidase activity (**f**) in Nipponbare and two *sag9* mutant seeds before or after aging. Data are presented as mean ± s.d of n = 3 replicates; statistical analysis were performed using One-way ANOVA versus Nipponbare within in each set of samples per time point in (**c–f**); ns, non-significant difference; singular asterisk and different letters indicate a statistically significant difference at *P* < 0.05; two asterisks, *P* < 0.01. DOD, days of deterioration; MA, months of natural aging.


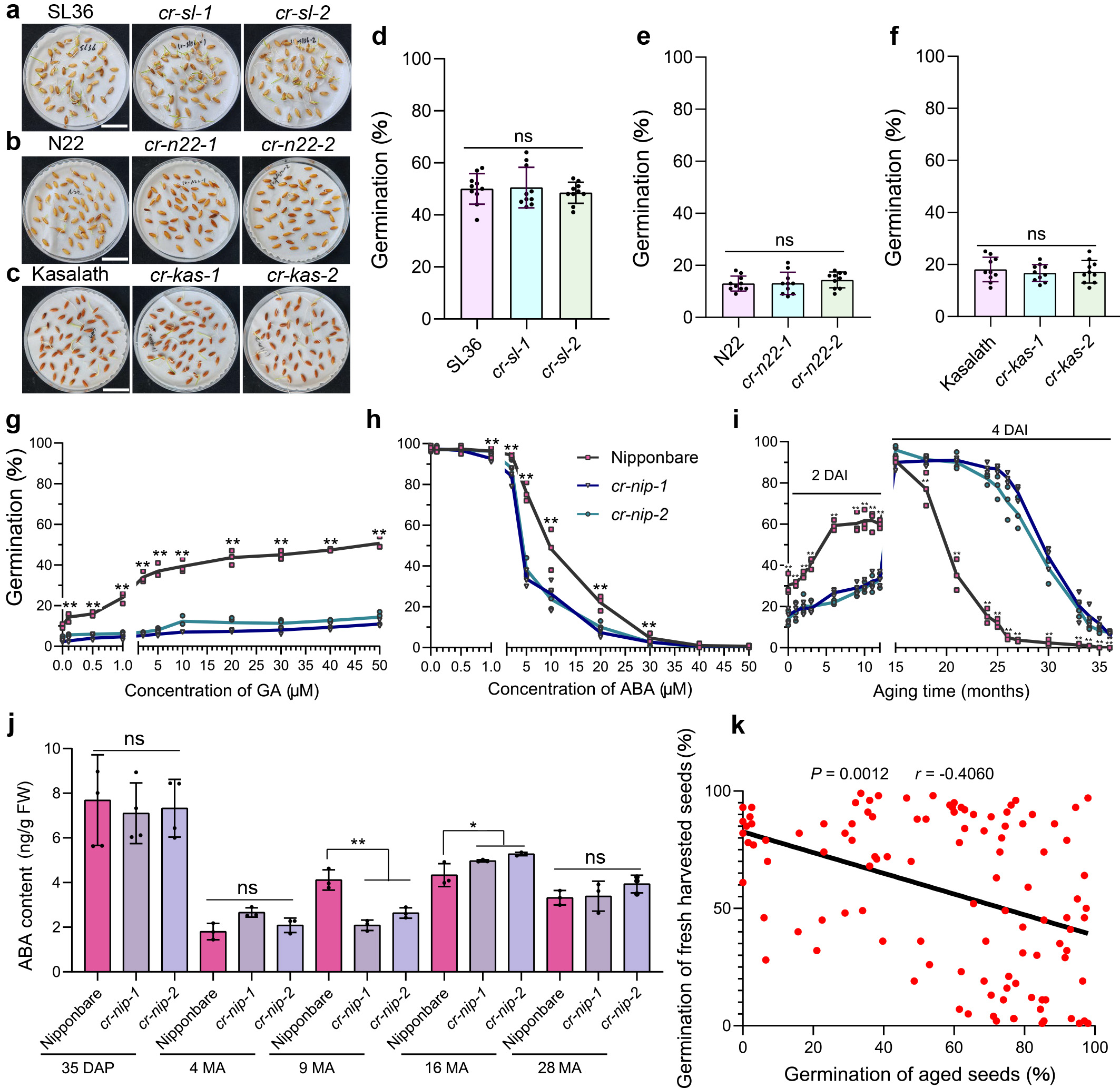


S**upplementary Fig. 9 *SAG9* confers Nipponbare differential seed vigor at the after-ripening and late-storage stages compared to *sag9* mutants.**

**a**,**b**,**c**, Germination performance of fresh-harvested wild type and *sag9* mutants in SL36 (**a**), N22 (**c**) and Kasalath (**e**) background. Seeds were harvested at 45 DAP. DAP, days after pollination. Scale bars: 2 cm. **d**,**e**,**f**, Germination of fresh-harvested wild type and *sag9* seeds in SL36 (**b**), N22 (**d**) and Kasalath (**f**) backgrounds. Data are presented as mean ± s.d of n = 10, each biological replicate comprised three independent technical replicates. **g**,**h**, Germination performance of Nipponbare and two *sag9* mutant seeds under increasing concentrations of GA_3_ (**g**) and ABA (**h**) treatments. Seeds harvested at 35 DAH were used in (**g**) to investigate the role of GA₃ in dormancy-breaking; and seeds were treated at 45℃ for one week before used for (**h**) to explore the inhibitory effect of ABA on seed germination. DAH, days after heading. Data are presented as mean ± s.d of n = 3 replicates, each biological replicate comprised three independent technical replicates. **i**, Seed germination curve for Nipponbare and two *sag9* mutants under ambient storage. Data are presented as mean ± s.d of n = 3, each biological replicate comprised three independent technical replicates. Data points for 0–10 months of storage were collected from seeds harvested in 2023 and those for 15–36 months of storage seeds were harvested in 2021. **j**, ABA content of Nipponbare and two *sag9* mutant seeds. Seeds used for 4 MA (**e**, **i**), 9 MA (**f**, **j**), 16 MA (**g**, **k**) and 28 MA (**h**, **l**) were harvested at Nanjing (2025), Hainan (2025), Nanjing (2024) and Nanjing (2023), respectively, and were stored under room temperature condition. DAP, days after pollination; MA, months of natural aging. Data are presented as mean ± s.d. **k**, Pearson's correlation coefficient for seed dormancy and storability (The raw data are provided in Supplementary Table **1**). X axis: germination of seeds after aging, y axis: germination of fresh seeds. Germination data of the same accessions in two-years aging assays (Supplementary Table **2**) and published dormancy data (Supplementary Table **3**) were used in this analysis. Statistical analysis was performed using one-way ANOVA followed Duncan’s new multiple range tests for mutants versus Nipponbare within each time point in (**g–j**), and One-way ANOVA versus respective wild types in (**d–f**); ns, non-significant difference; one asterisk, *P* < 0.05; two asterisks, *P* < 0.01.


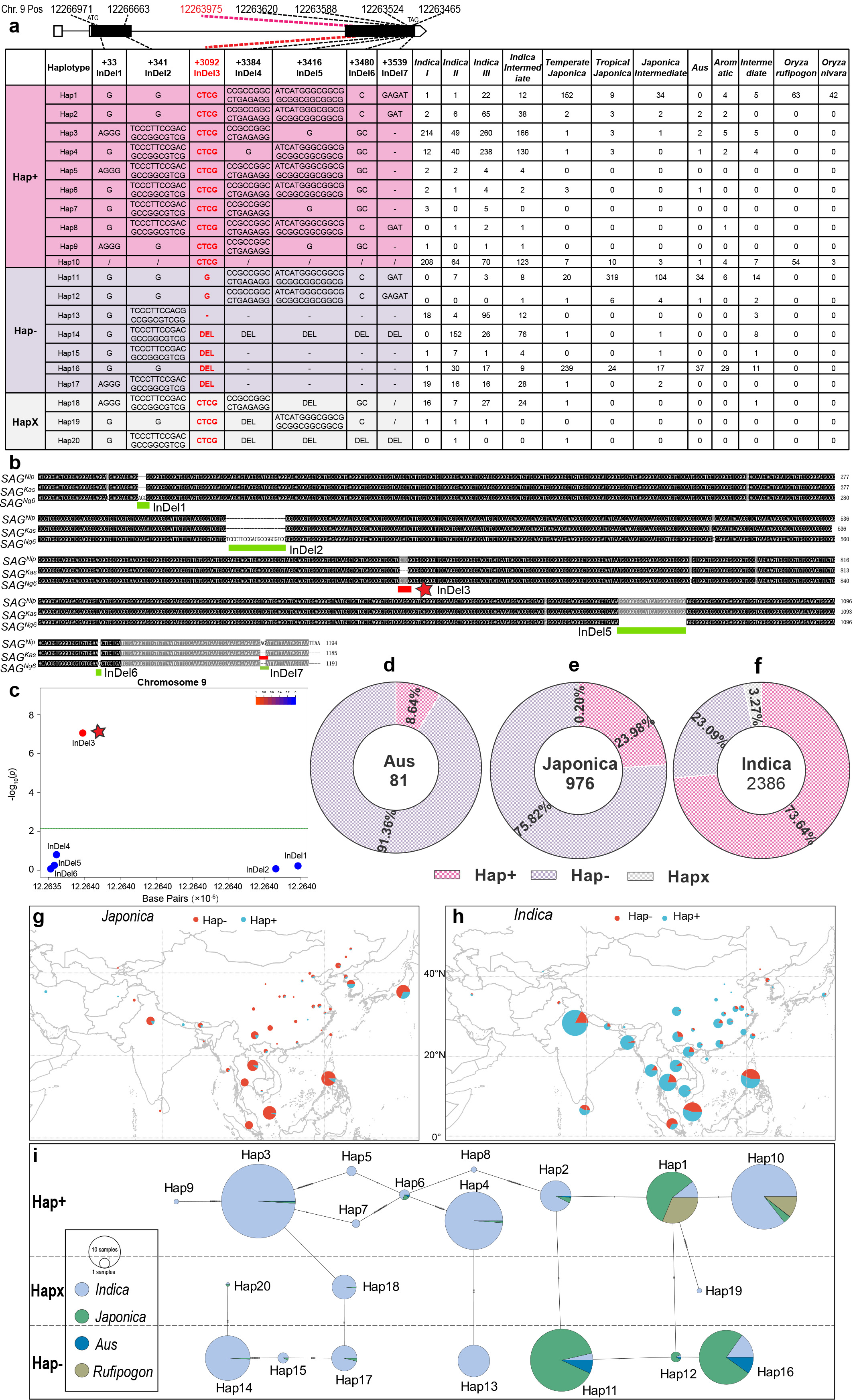


S**upplementary Fig. 10 Allelic distribution of *SAG9*.**

**a**, *SAG9* haplotype classification and frequency distribution in the 3,000 Rice Genomes Project [2-4] and 162 sequenced wild rice accessions [5]. Haplotypes were classified based on seven InDels in the *SAG9* coding region. Haplotypes for the 3K rice population are detailed in Supplementary Table **4** and those of wild rice are detailed in Supplementary Table **5**. ‘–’ indicates the translation termination codon appears before this InDel, ‘/’ indicates that the InDel at this position is not involved in the haplotype classification, ‘DEL’ indicates that the InDel type at this position is a deletion, and numbers indicate the number of accessions. **b**, Alignment of the *SAG9* coding sequence between Nipponbare (*SAG9^Nip^*), Kasalath (*SAG9^Kas^*) and Ninggeng6 (*SAG9^Ng6^*). Black shading represents highly conserved nucleotide sequences, green lines indicate InDels between Nipponbare and Ninggeng6, red lines indicate InDels between Nipponbare and Kasalath, the star indicates the major natural variant affecting function. **c**, *SAG9*-GWAS genome-wide association study based on InDel1–InDel6 in the coding sequence and storability of 691 accessions in the rice 3K project. The red star represents the major InDel related to seed storability. **d–f**, Distribution of *SAG9* *Hapx*, *Hap-* and *Hap+* haplotypes in rice subgroups *Aus* (**d**), *Japonica* (**e**) and *Indica* (**f**). **g**,**h**, Geographic distribution of *Hap-* and *Hap+* in the *Japonica* subgroup (**g**) and *Indica* subgroup (**h**). N, north latitude. Circle size represents the number of accessions and the geographical information of the accessions is from 3,000 Rice Genomes Project [2-4]. **i**, *SAG9* haplotype network based on InDels in the coding region using 3,000 Rice Genomes Project (Supplementary Table **4**) and 162 sequenced wild rice accessions (Supplementary Table **5**). Each pie chart represents one haplotype and the area of the pie chart is proportional to the haplotype frequency. Each pie chart is further divided based on different rice subgroups within that haplotype and each segment on the line connecting different pies represents one mutation.


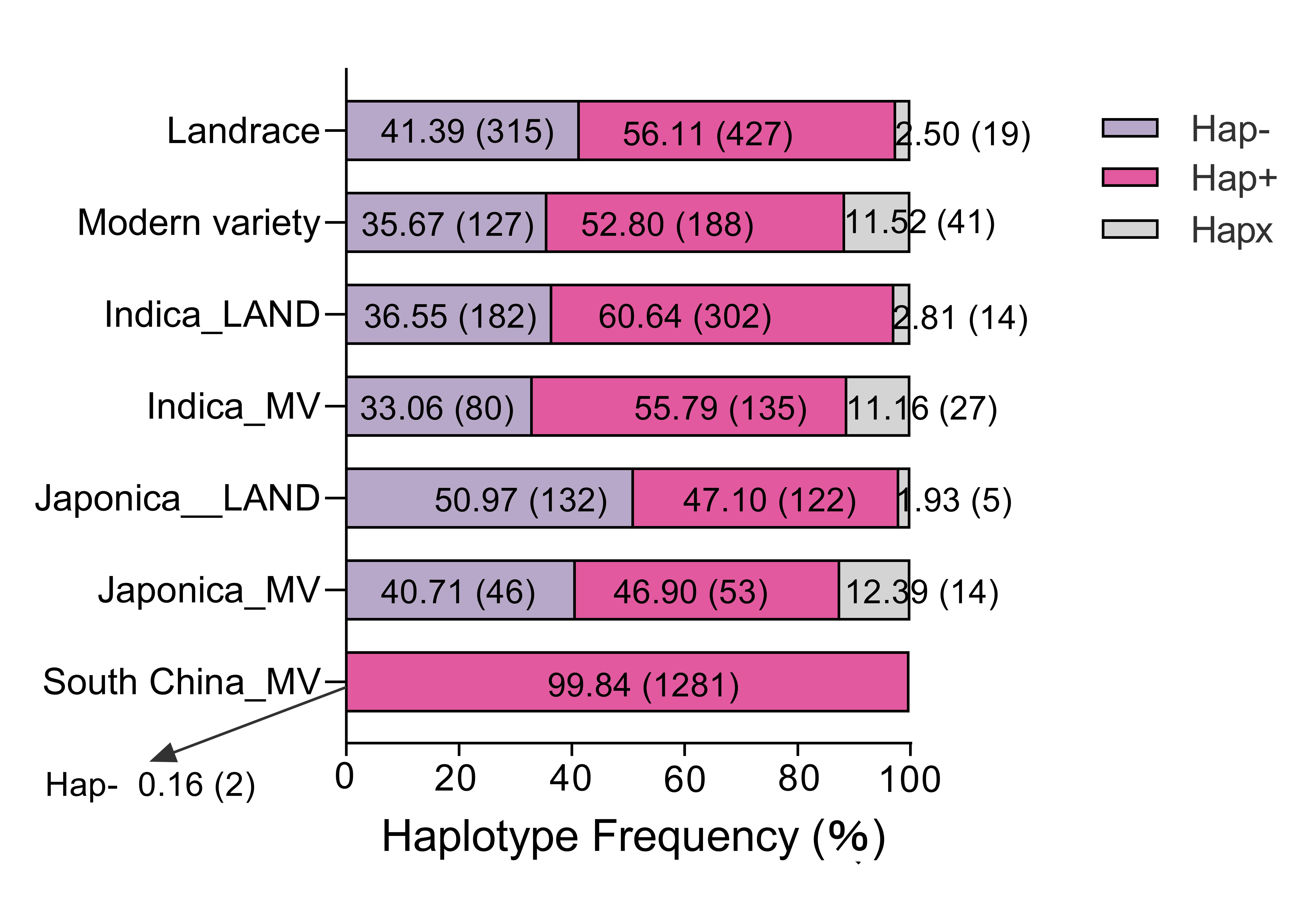


**Supplementary Fig. 11 *SAG9* haplotype distribution in landraces and modern varieties.**

Indica_LAND: Landrace of *Indica* subgroup, Indica_MV: Modern variety of *Indica* subgroup, Japonica_LAND: Landrace of *Japonica* subgroup, Japonica_MV: Modern variety of *Japonica* subgroup, South China_MV: Modern variety from southern China. The numbers indicate the proportion of the haplotype and the numbers in parentheses indicate the number of accessions. Landraces and modern varieties are detailed in Supplementary Table **4,** landraces and modern varieties from southern China are detailed in Supplementary Table **8**.


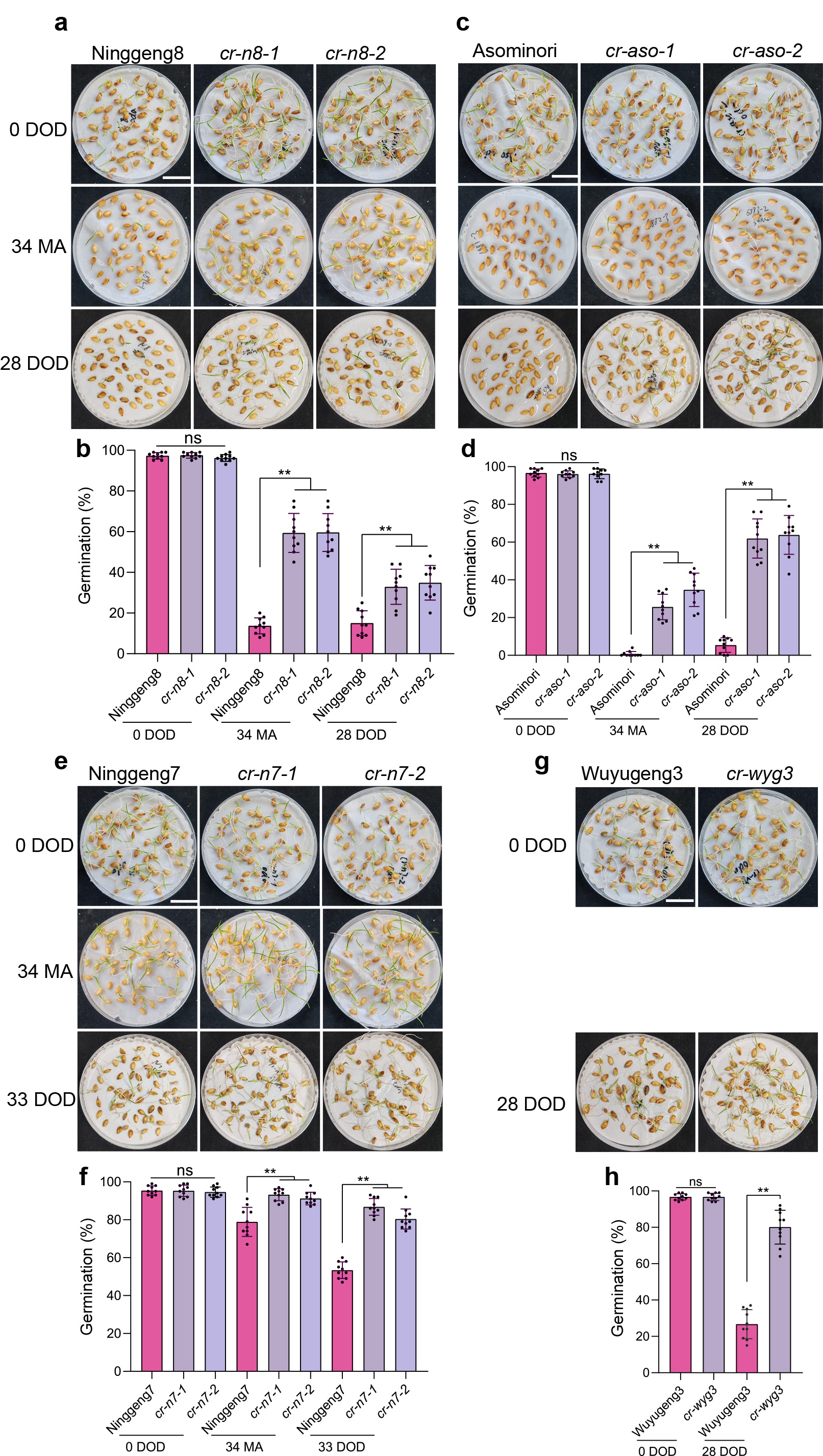


**Supplementary Fig. 12 Mutation of *SAG9* in select modern rice varieties improves seed storability.**

**a**,**b**,**e**,**g**, Germination performance of wild type and respective *sag9* mutants in Ninggeng8 (**a**), Asominori (**c**), Ninggeng7 (**e**) and Wuyugeng3 (**g**) backgrounds before or after aging. **b**,**d**,**f**,**h**, Germination of wild type and respective *sag9* mutants in Ninggeng8 (**b**), Asominori (**d**), Ninggeng7 (**f**) and Wuyugeng3 (**h**) backgrounds before or after aging. DOD, days of deterioration; MA, months of natural aging. Photographs were taken 7–10 d after imbibition. Scale bars: 2 cm. Data are presented as mean ± s.d of n = 10. Each biological replicate comprised three independent technical replicates. Statistical analysis was performed using one-way ANOVA followed Duncan’s new multiple range tests for mutants versus respective wild types within each time point in (**b**, **d** and **f**) and two-tailed Student's *t* tests within each time point in (**h**); ns, non-significant difference; done asterisk and different letters indicate a statistically significant difference at *P* < 0.05, two asterisks, *P* < 0.01.


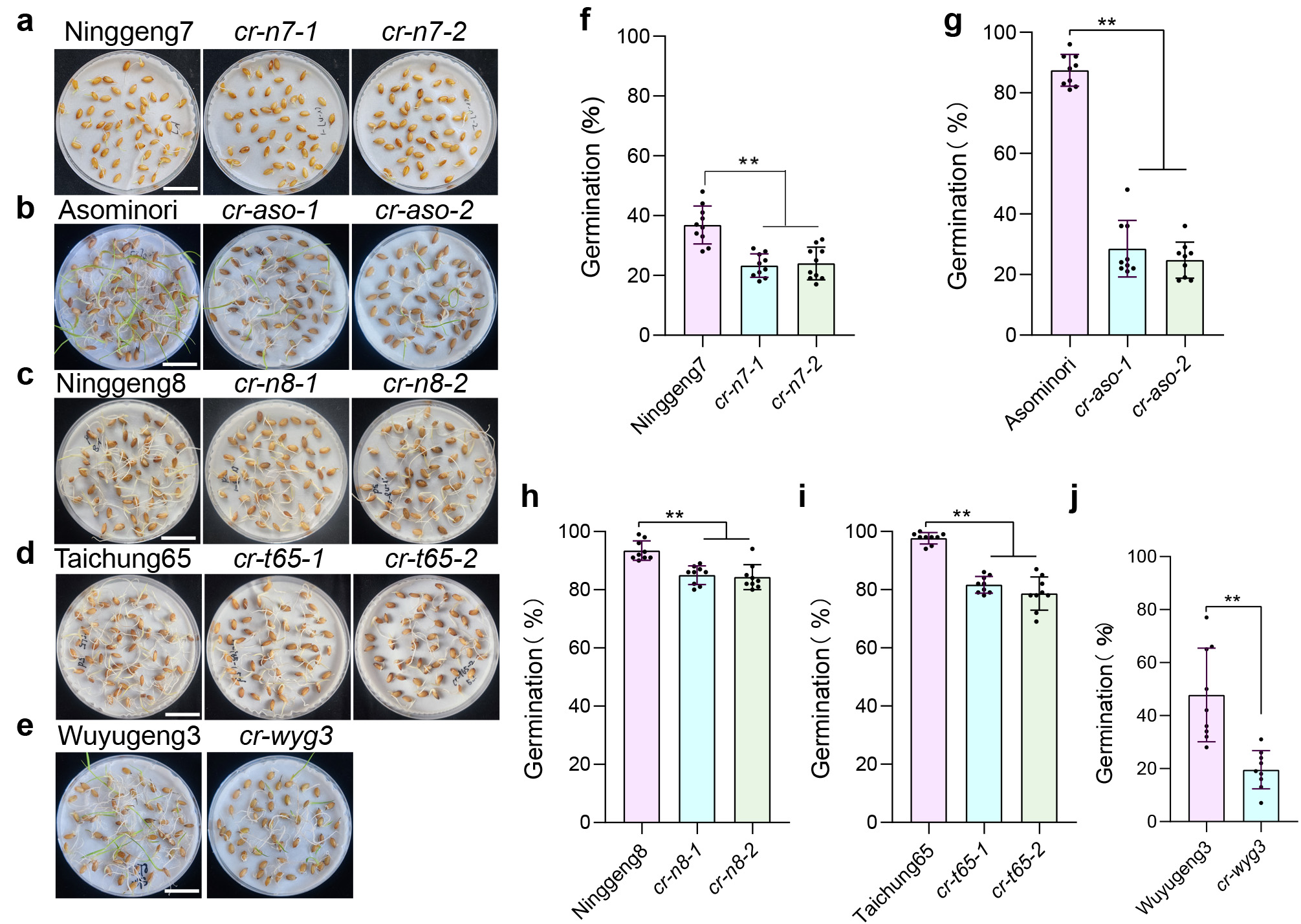


**Supplementary Fig. 13 Knockout of *SAG9* improves dormancy in select modern rice varieties with diverse haplotypes.**

**a–e**, Germination performance of fresh harvested wild type and respective *sag9* mutants in Ninggeng7 (**a**), Asominori (**b**), Ninggeng8 (**c**), Taichung65 (**d**) and Wuyugeng3 (**e**) backgrounds. Scale bars: 2 cm. **f**–**j**, Germination quantification of fresh harvested wild type and respective *sag9* mutants in Ninggeng7 (**f**), Asominori (**g**), Ninggeng8 (**h**), Taichung65 (**i**) and Wuyugeng3 (**j**) backgrounds. Ninggeng7 and mutant seeds were harvested at 50 DAH and at 35 DAH for Asominori, Ninggeng8, Taichung65 and Wuyugeng3 backgrounds and respective mutants. Photographs were taken 7–10 d after imbibition. Data are presented as mean ± s.d of 10 replicates. Each biological replicate comprised three independent technical replicates. Statistical analysis was performed using one-way ANOVA followed Duncan’s new multiple range tests versus controls in (**f**–**i**) and two-tailed Student's *t* test in (**j**); one asterisk and different letters indicate a statistically significant difference at *P* < 0.05, two asterisks, *P* < 0.01.


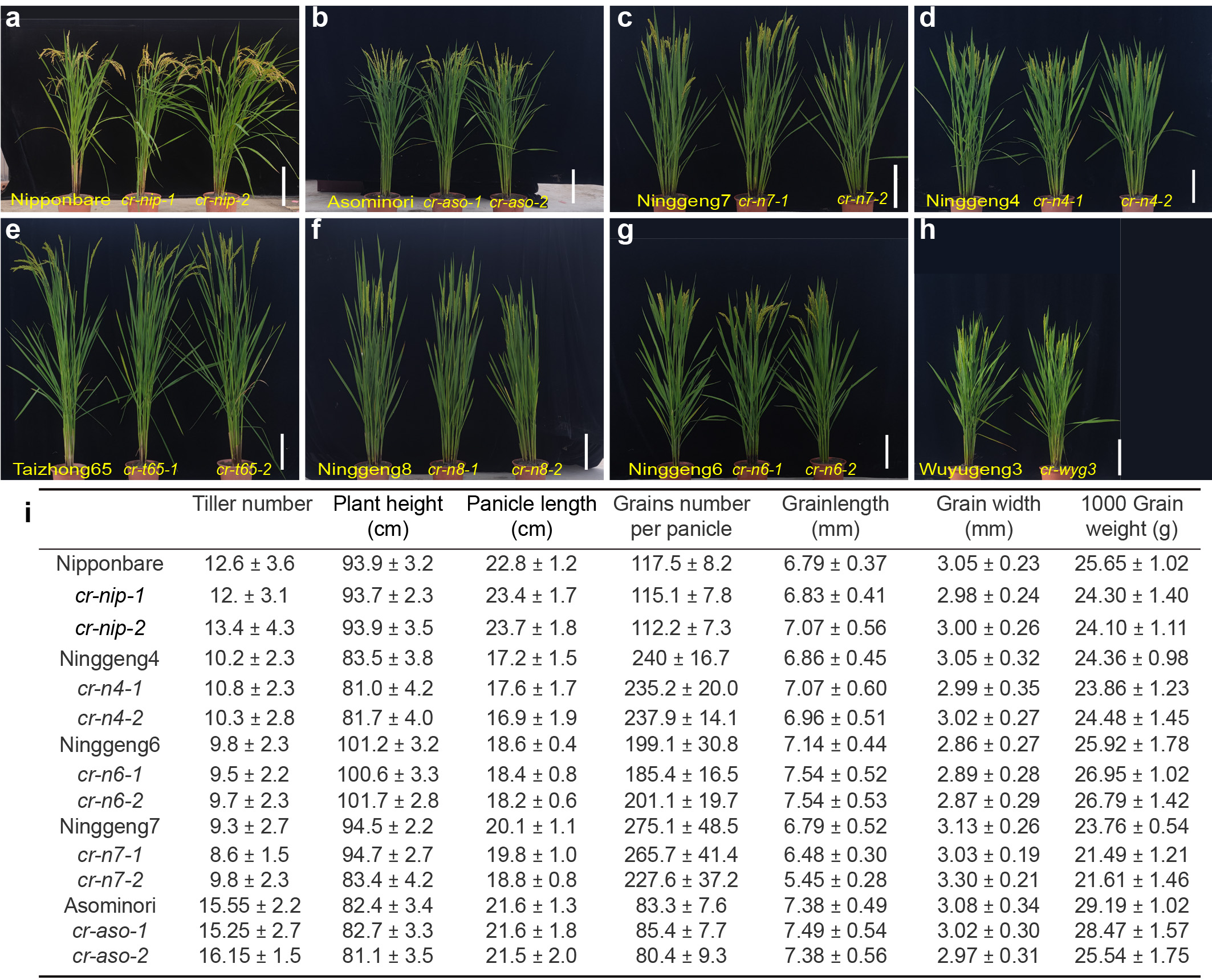


**Supplementary Fig.14 *SAG9* knockout lines showed no difference in main agronomic traits with WT.**

**a**–**h**, Representative shoot phenotypes of wild type and respective *sag9* mutants in Nipponbare (**a**), Asominori (**b**), Ninggeng7 (**c**), Ninggeng4 (**d**), Taichung65 (**e**), Ninggeng8 (**f**), Ninggeng6 (**g**), and Wuyugeng3 (**h**) backgrounds. Scale bar = 20 cm. **i**, Tiller number, plant height, panicle length, grain-number per panicle, grain length, grain width and 1,000 grain weight of wild type and respective *sag9* mutants as shown in (**a**–**h**). Data are shown as mean value with s.d of n = 20 replicates. Note: Occasionally, the agronomic traits of *SAG9* mutant lines were found better or worse than WT, but no consistent trend observed between independent lines or independent backgrounds, probably because of the occasional off-target or callus-induced mutations. Thus, we draw the conclusion that knockout of *SAG9* only affects seed storability and dormancy but not the other agronomic traits*.*


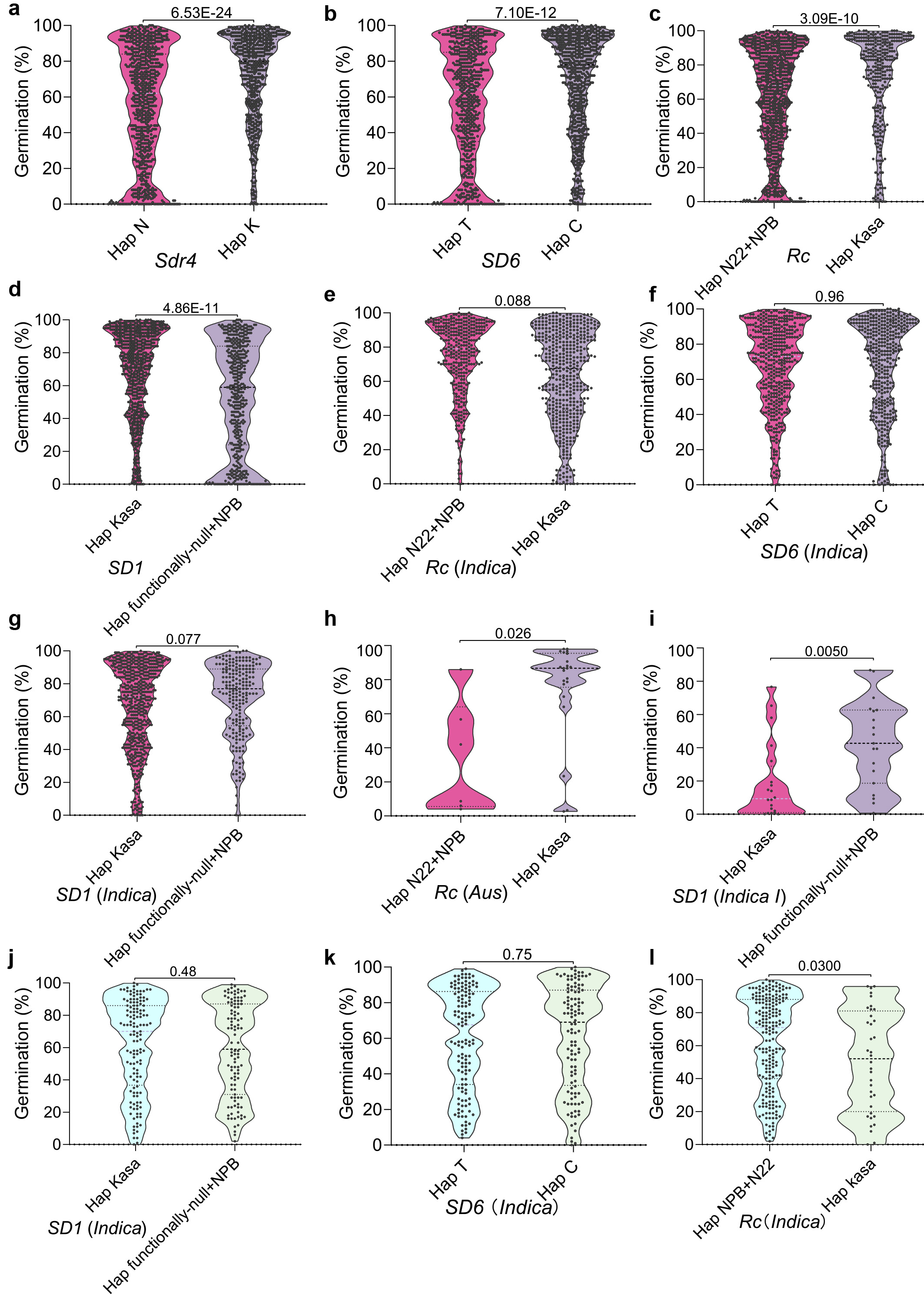


**Supplementary Fig. 15 Seed-dormancy-related genes also regulate seed storability.**

**a–d**, Germination of aged seeds for *Sdr4* strong seed-dormancy haplotype *Hap K* and weak haplotype *Hap N* (**a**), between *SD6* strong seed-dormancy haplotype *Hap C* and weak haplotype *Hap T* (**b**), between *Rc* strong seed-dormancy haplotype *Hap Kasa* and weak haplotype *Hap N22 + NPB* (**c**), between *SD1* strong seed-dormancy haplotype *Hap functionally-null + NPB* and weak haplotype *Hap Kasa* (**d**). **e–g**, Germination percentage of aged seeds with different haplotypes of *Rc* (**e**), *SD6* (**f**) and *SD1* (**g**) in *Indica*. Two years of aging data for 691 varieties were used in (**a–g**) and are provided in Supplementary Table **2**. **h**,**i**, Germination percentage of aged seeds with different haplotypes of *Rc* (**h**) and *SD1* (**i**). To eliminate the influence of genetic heterogeneity, only *Aus* accessions were used in (**h)** and only *Indica Ⅰ* accessions (*SDR3.1* and *GH3-2* mainly elite haplotypes; *Sdr4* and *SAG9* mainly weak haplotypes) were used in (**i)**. Published seed-storability data were used in (**h**,**i**) and are provided in Supplementary Table **6**. **j–l**, Germination percentage of *SD1* (**j**), *SD6* (**k**) and *Aus* (**l**) different haplotypes with fresh harvested seeds in *Indica*. Published seed-dormancy data were used in (**j–l**) and are provided in Supplementary Table **3**. Haplotype classification was based on previous studies [6-8]. Dotted lines denote the 25^th^ percentile, the median and the 75^th^ percentile. Statistical analysis was performed using two-tailed Student's *t* tests and exact *P* values are marked for each comparison.


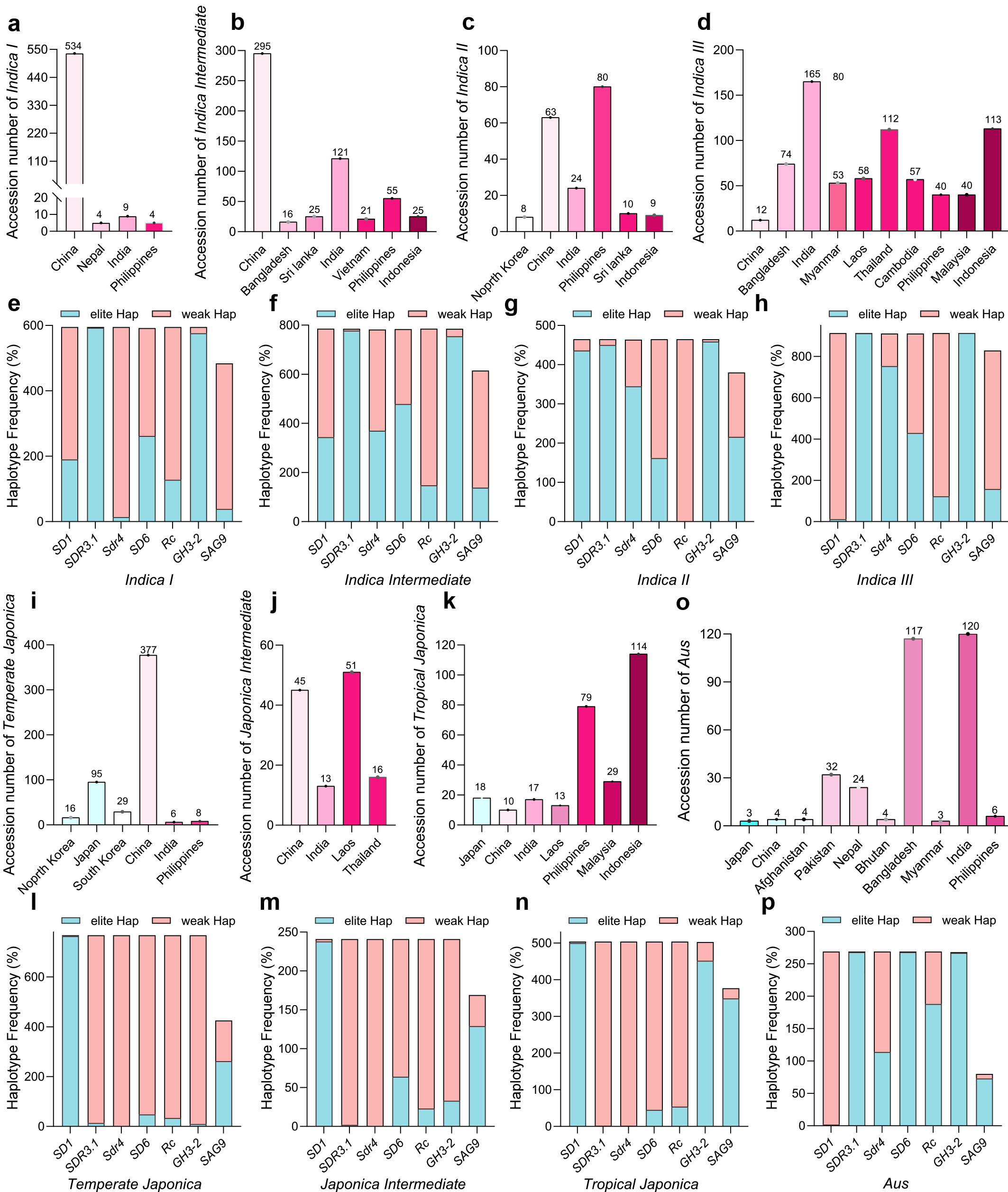


**Supplementary Fig. 16 Allelic distribution of rice *SAG9, SD1, SDR3.1, Sdr4, SD6, Rc* and *GH3-2* haplotypes.**

**a–d**,**i–k**,**o**, Geographic distribution of rice subgroups *Indica I* (**a**), *Indica* Intermediate (**b**), *Indica II* (**c**), *Indica Ⅲ* (**d**), Temperate *Japonica* (**i**), *Japonica* Intermediate (**j**), Tropical *Japonica* (**k**), and *Aus* (**o**) generated using data from the 3,000 Rice Genomes Project [2-4]. Accessions are provided in Supplementary Table **4**. **e–h**,**l–n**,**p**, Haplotype frequency for *SAG9*, *SD1*, *SDR3.1*, *Sdr4*, *SD6*, *Rc* and *GH3-2* in rice subgroups *Indica I* (**e**), *Indica* Intermediate (**f**), *Indica II* (**g**), *Indica Ⅲ* (**h**), Temperate *Japonica* (**l**), *Japonica* Intermediate (**m**), Tropical *Japonica* (**n**) and *Aus* (**p**). Elite Hap: strong seed-storability or dormancy haplotypes, Weak Hap: weak seed-storability or dormancy haplotypes. Haplotype classification was based on [6-9] and accessions are provided in Supplementary Table **4**.


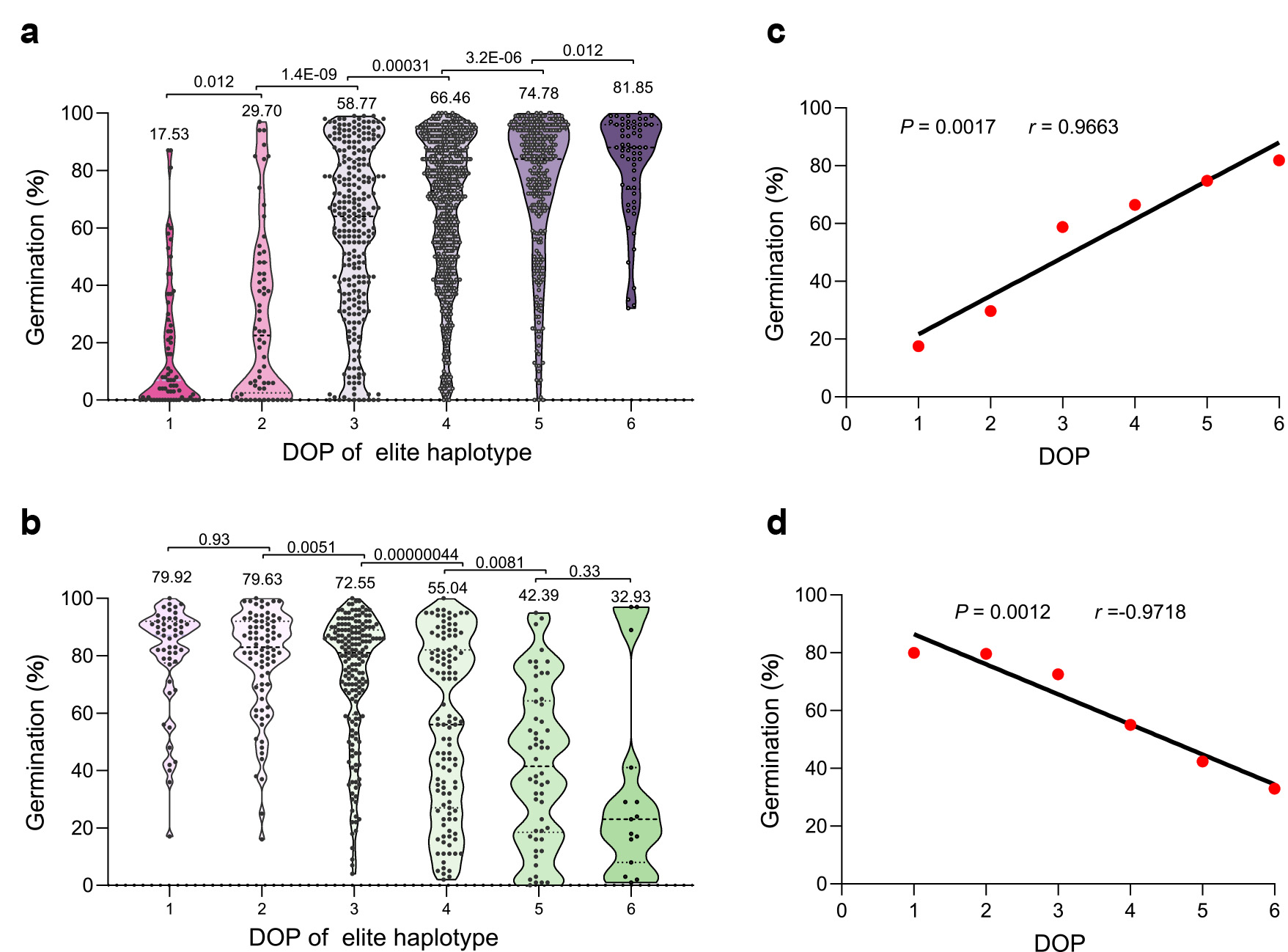


**Supplementary Fig. 17 The degree of pyramiding of elite haplotypes positively correlates with rice seed storability and dormancy.**

**a**,**b**, Germination percentage of aged (**a**) and fresh harvested seeds (**b**) in accessions with different degrees of pyramiding (DOP) of elite alleles. Two years of artificial-aging data for 691 varieties were used in (**a**) (Supplementary Table **2**) and published dormancy data were used in (**b**) (Supplementary Table **3)**. X axis: DOP of *SAG9*, *SD1*, *SDR3.1*, *Sdr4*, *SD6*, *Rc* and *GH3-2* elite haplotypes. Dotted lines denote the 25^th^ percentile, the median and the 75^th^ percentile. Statistical analysis was performed using one-way ANOVA followed Duncan’s new multiple range tests, numbers above violin plots are average germination percentages, numbers above bars are the exact *P* values. **c**,**d**, Pearson's correlation coefficients for DOP of elite haplotypes with seed storability (**c**) and dormancy (**d**).


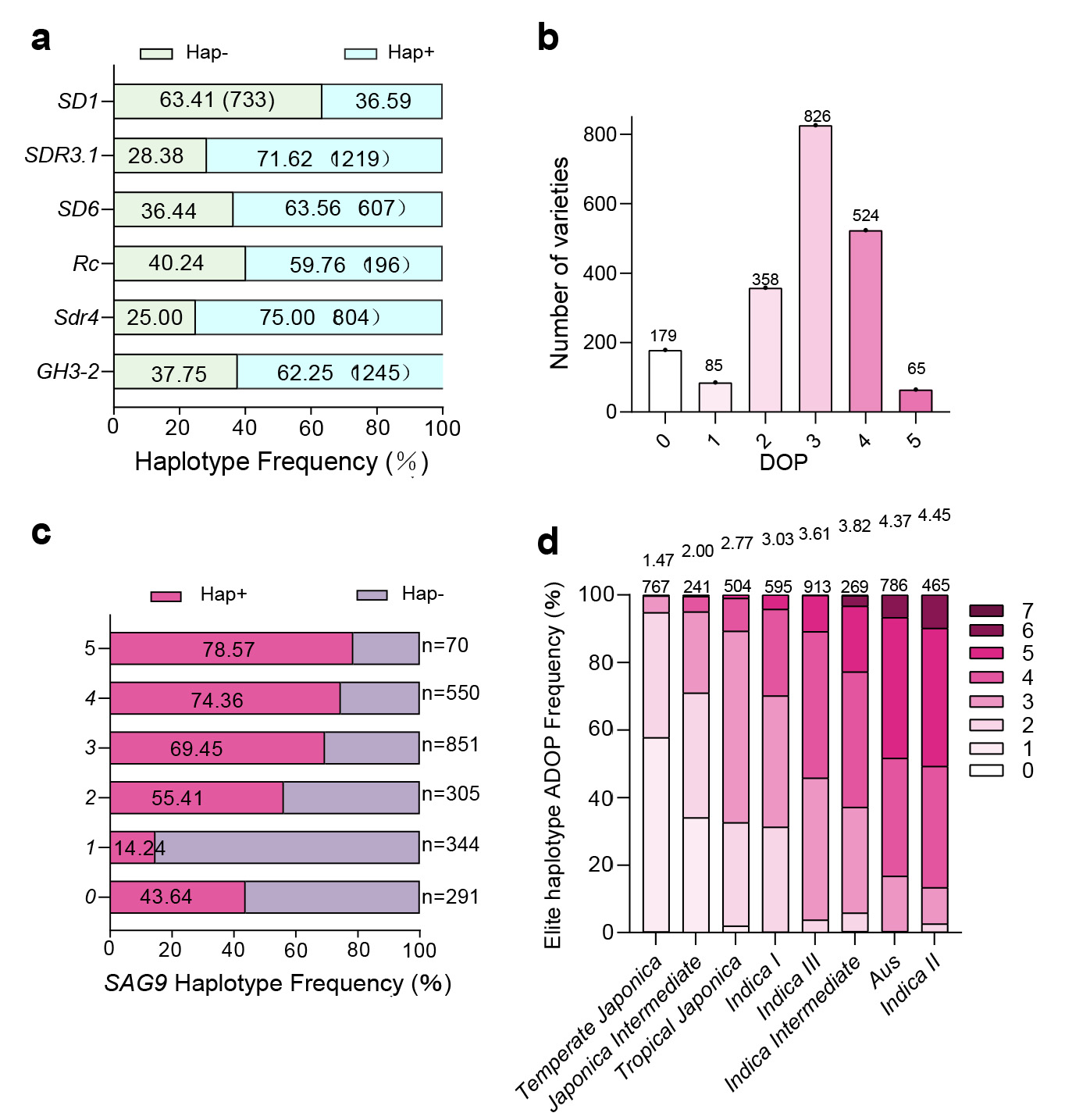


**Supplementary Fig. 18 *SAG9* *Hap+* shows linkage in rice varieties with elite haplotypes of *SDR3.1*, *Sdr4*, *SD6*, *Rc* and *GH3-2*.**

**a**, Frequency of *SAG9* haplotypes *Hap+* and *Hap-* in *SD1*, *SDR3.1*, *SD6*, *Rc*, *Sdr4* or *GH3-2* elite-haplotype backgrounds. The numbers indicate the proportion of the haplotype and the numbers in parentheses indicate the number of accessions examined. **b**, Frequency of DOP for *SDR3.1/Sdr4/SD6/Rc/GH3-2* elite haplotypes in the *SAG9* *Hap+* background. **c**, Frequency of *SAG9* haplotypes *Hap+* and *Hap-* in different DOP of *SDR3.1*, *SD6*, *Rc*, *Sdr4* and *GH3-2* elite haplotypes. Numbers indicate the proportion of *SAG9 Hap+*. **d**, Average DOP (ADOP) of rice subgroups. Darker box shading indicates larger DOP values, box size represents the frequency of each DOP accessions, numbers above the column re the subgroup size; numbers along the top are the average DOP. The varieties pyramided with 6 or all 7 elite haplotypes are provided in Supplementary Table **9**. Information of subgroups and haplotypes are in Supplementary Table **4**.


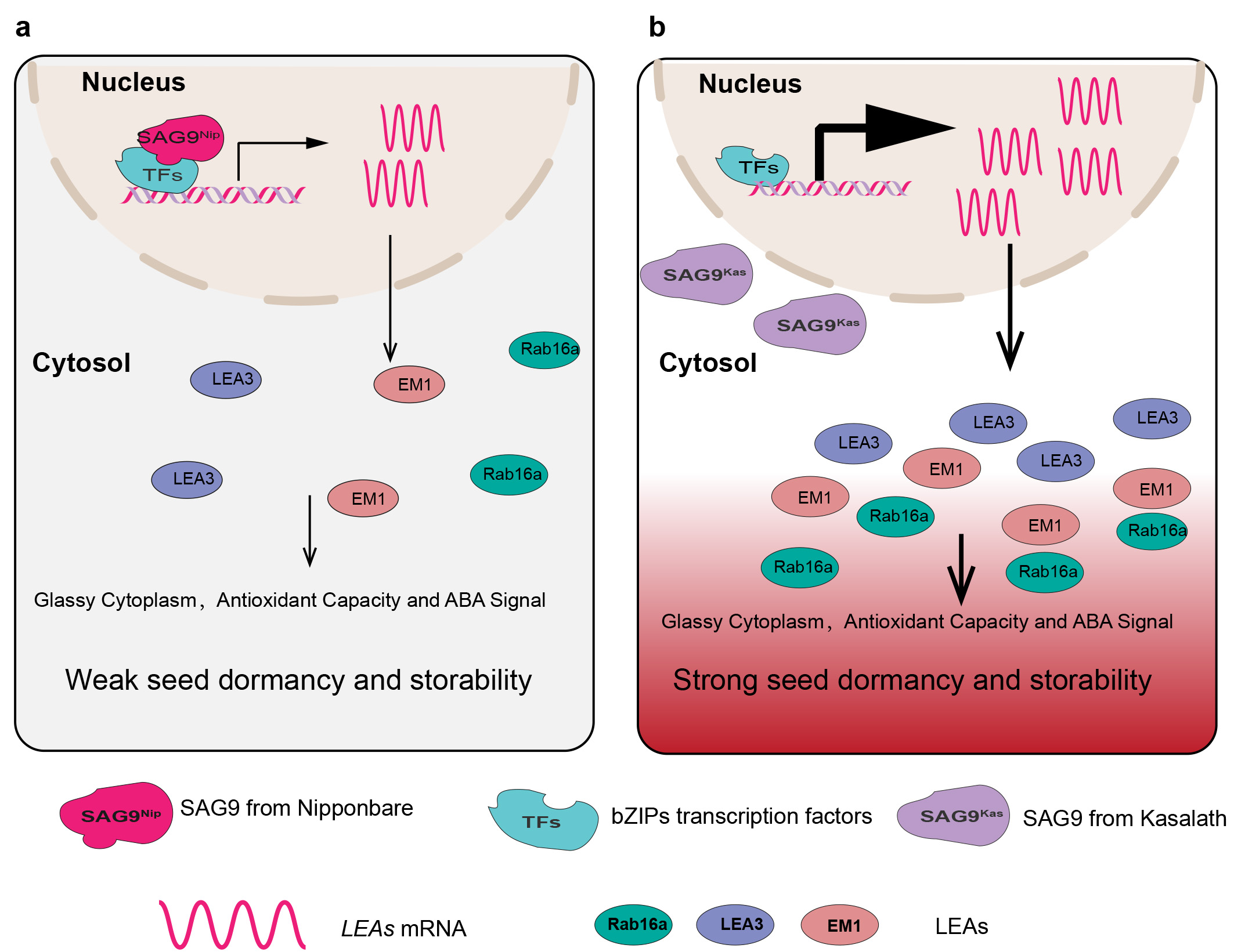


**Supplementary Fig. 19 Proposed model for rice SAG9** **regulation of seed dormancy and storability.**

**a**,**b**, Models of SAG9 regulating rice seed dormancy and storability in Nipponbare (**a**) and Kasalath (**b**). *EM1*, *LEA3* and *Rab16A* encode LEA proteins and serve as marker genes for ABA signal transduction. Their accumulation facilitates the formation of glassy cytoplasm and endows cells with antioxidant capacity, which in turn slows the processes of dormancy-breaking and seed-aging. bZIPs are core transcription factors in ABA signal transduction; they can directly bind to the promoters of the aforementioned *LEAs* to promote their expression, thereby positively regulating seed dormancy and storability. In Nipponbare (**a**), SAG9^Nip^ localizes to the nucleus and interacts with these bZIPs, inhibiting their activation effect on downstream *LEAs* and consequently reducing seed dormancy and storability. This inhibitory effect is absent in Kasalath (**b**), due to the extranuclear localization of SAG9^Kas^ and the loss of its interaction with bZIPs. As a result, Kasalath exhibits stronger seed dormancy and storability.


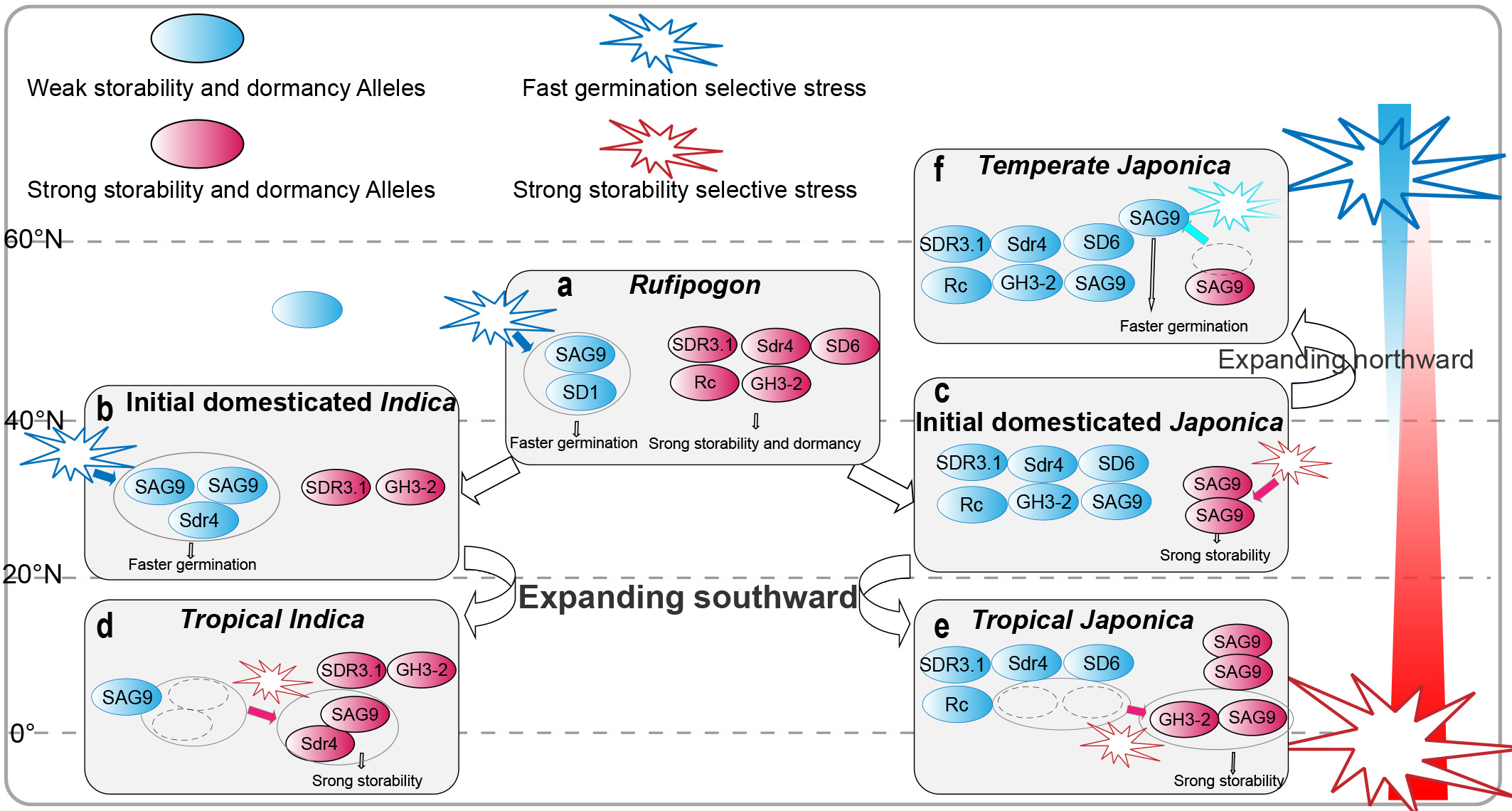


**Supplementary Fig. 20 *SAG9* acts as an equalizer and major target in the domestication of rice seed dormancy or storability.**

**a–f**, Genotype of *SAG9*, *SD1*, *SDR3.1*, *Sdr4*, *SD6*, *Rc* and *GH3-2* in wild rice (**a**), initial domesticated *Indica* (**b**), Tropical *Indica* (**c**), Initial domesticated *Japonica* (**d**), Temperate *Japonica* (**e**) and Tropical *Japonica* (**f**). In wild rice, nearly all varieties harbor *SDR3.1*, *Sdr4*, *SD6*, *Rc* and *GH3-2* elite haplotypes as well as *SD1* and *SAG9* weak-dormancy haplotypes [6-9]. In *Indica*, nearly all varieties harbor *SDR3.1* and *GH3-2* elite haplotypes, endowing seeds with longer longevity and dormancy, therefore more *SAG9 Hap+* was selected (Supplementary Fig. **16e–h**) probably for faster germination. From *Indica* *I* to *Indica* *III* subgroups, the geographical distribution of accessions changed from main temperate and semi-tropical regions (e.g. China) to main tropical areas (e.g. Philippines, Thailand, Malaysia and Indonesia) (Supplementary Fig. **16a–d**), and the frequency of *SAG9* and *Sdr4* elite haplotypes shows an increasing trend (Supplementary Fig. **16e–h**), which is likely an adaptation to the increase in temperature and humidity. In *Japonica*, *SDR3.1*, *Sdr4*, *SD6* and *Rc* (except *SD1*) mainly harbored weak storability haplotypes (Supplementary Fig. **16l–n**), giving seeds shorter longevity, therefore *SAG9* *Hap-* was strongly selected for longer longevity. From the Temperate *Japonica* subgroup to the Tropical *Japonica* subgroup, the geographic distribution of accessions shifts from major temperate regions (e.g. North Korea, South Korea, Japan and China) to tropical areas (e.g. India, Philippines, Laos, Malaysia and Indonesia) (Supplementary Fig. **16i–k**), and the frequency of *SAG9* and *GH3-2* elite haplotypes increased gradually (Supplementary Fig. **16l–n**), likely as an adaptation to elevated temperature and humidity, especially for Tropical *Japonica* since nearly all accessions harbor *SAG9* and *GH3-2* elite haplotypes (Supplementary Fig. **16n**). Overall, *SAG9* serves as an equalizer and major target in the domestication of seed vigor. *SDR3.1*, *Sdr4*, *SD6* and *Rc* weak haplotypes were preferred in *Japonica* that caused strong-storability selective stress in *SAG9* and many *Hap-* were selected, especially in Tropical *Japonica*. *GH3-2*, *SDR3.1*, *Sdr4*, *SD6* and *Rc* elite haplotypes were selected in *Indica* causing enhanced fast-germination selective stress in *SAG9* and many *Hap+* were selected. These observations reflect the antagonistic effects between SAG9 and other factors during the domestication of seed vigor. Under strong strong-storability selective stress in tropical areas, *Sdr4* and *GH3-2* elite haplotypes were selected alongside *SAG9 Hap-* in *Indica* and *Japonica*, respectively, which reflects the synergistic effects of *SAG9* with *Sdr4* and *GH3-2* during the domestication of seed vigor.

**Supplementary References**

1. He Y, Zhang T, Sun H *et al.* A reporter for noninvasively monitoring gene expression and plant transformation. *Hortic Res*. 2020; **7**(1): 152.

2. Wang W, Mauleon R, Hu Z *et al.* Genomic variation in 3,010 diverse accessions of Asian cultivated rice. *Nature*. 2018; **557**(7703): 43-49.

3. Li JY, Wang J, Zeigler RS. The 3,000 rice genomes project: new opportunities and challenges for future rice research. *Gigascience*. 2014; **3**: 8.

4. Zhao H, Yao W, Ouyang YD *et al.* RiceVarMap: a comprehensive database of rice genomic variations. *Nucleic Acids Research*. 2015; **43**(D1): D1018-D1022.

5. Zheng X, Pang H, Wang J *et al.* Genomic signatures of domestication and adaptation during geographical expansions of rice cultivation. *Plant Biotechnol J*. 2022; **20**(1): 16-18.

6. Guo N, Tang S, Wang Y *et al.* A mediator of OsbZIP46 deactivation and degradation negatively regulates seed dormancy in rice. *Nat Commun*. 2024; **15**(1): 1134.

7. Zhao B, Zhang H, Chen T *et al.* *Sdr4* dominates pre-harvest sprouting and facilitates adaptation to local climatic condition in Asian cultivated rice. *J Integr Plant Biol*. 2022; **64**(6): 1246-1263.

8. Xu F, Tang J, Wang S *et al.* Antagonistic control of seed dormancy in rice by two bHLH transcription factors. *Nat Genet*. 2022; **54**(12): 1972-1982.

9. Yuan Z, Fan K, Wang Y *et al.* *OsGRETCHENHAGEN3-2* modulates rice seed storability via accumulation of abscisic acid and protective substances. *Plant Physiol*. 2021; **186**(1): 469-482.
